# Identification of Rhythmically Expressed LncRNAs in the Zebrafish Pineal Gland and Testis

**DOI:** 10.3390/ijms22157810

**Published:** 2021-07-22

**Authors:** Shital Kumar Mishra, Taole Liu, Han Wang

**Affiliations:** 1 Center for Circadian Clocks, Soochow University, Suzhou 215123, China; mishrasz@suda.edu.cn (S.K.M.); liutaole18@163.com (T.L.); 2 School of Biology & Basic Medical Sciences, Medical College, Soochow University, Suzhou 215123, China

**Keywords:** noncoding RNAs, lncRNAs, lncRNA-encoded peptides, circadian rhythmicity, zebrafish, bioinformatics

## Abstract

Noncoding RNAs have been known to contribute to a variety of fundamental life processes, such as development, metabolism, and circadian rhythms. However, much remains unrevealed in the huge noncoding RNA datasets, which require further bioinformatic analysis and experimental investigation—and in particular, the coding potential of lncRNAs and the functions of lncRNA-encoded peptides have not been comprehensively studied to date. Through integrating the time-course experimentation with state-of-the-art computational techniques, we studied tens of thousands of zebrafish lncRNAs from our own experiments and from a published study including time-series transcriptome analyses of the testis and the pineal gland. Rhythmicity analysis of these data revealed approximately 700 rhythmically expressed lncRNAs from the pineal gland and the testis, and their GO, COG, and KEGG pathway functions were analyzed. Comparative and conservative analyses determined 14 rhythmically expressed lncRNAs shared between both the pineal gland and the testis, and 15 pineal gland lncRNAs as well as 3 testis lncRNAs conserved among zebrafish, mice, and humans. Further, we computationally analyzed the conserved lncRNA-encoded peptides, and revealed three pineal gland and one testis lncRNA-encoded peptides conserved among these three species, which were further investigated for their three-dimensional (3D) structures and potential functions. Our computational findings provided novel annotations and regulatory mechanisms for hundreds of rhythmically expressed pineal gland and testis lncRNAs in zebrafish, and set the stage for their experimental studies in the near future.

## 1. Introduction

Long noncoding RNAs (lncRNAs), lacking protein coding abilities as their names imply, are a special class of the transcribed RNAs with a length longer than 200 nucleotide base pairs [1]. Tens of thousands of functional lncRNAs [2] have already been catalogued from numerous organisms, including humans [3,4]. Although lncRNAs do not encode functional proteins, they regulate a diverse set of biological processes such as genomic transcription [5], gene regulation [6], cell fate determination [7], development of the nervous system, muscle performance [8], and human diseases [9]. Intriguingly, several lncRNAs have recently been confirmed [10] to encode micropeptides (also known as microproteins), which are the polypeptides containing < 100 amino acids [11], distinguishable from the canonical proteins made of about 400 or more amino acids [12]. For example, the lncRNA-encoded micropeptide Toddler (also known as Elabela, Ende, linc_tmem192) promotes cell movement by regulating apelin receptors in zebrafish [13]. A study found a conserved micropeptide myoregulin encoded by the skeletal-muscle-specific lncRNA [8]. Another study identified a 60-amino=acid polypeptide ASRPS encoded by the lncRNA LINC00908, which contained sORFs [14,15]. A recent study identified the lncRNA MIR155HG, which encoded miPEP155—a 17-amino-acid micropeptide involved in antigen trafficking [10]. A recent study identified several lncRNA-encoded micropeptides implicated in tumorigenesis [16]. These findings have inspired biologists to identify novel lncRNA-encoded peptides and investigate their regulatory functions.

Intriguingly, lncRNAs also contribute to circadian regulation [17,18]. For example, a recent study [17] found the role of lncRNAs in perturbing the circadian rhythms of hepatoma cells. Another study [19] found differential expression of over 100 lncRNAs in the rat pineal gland—the tissue directly responsible for synthesizing the hormone melatonin [20]. However, very little is known about lncRNA-mediated clock regulation. The testis is an important organ involved in several biological functions, such as producing gametes and circulating androgens [21]. The germ cells divide and differentiate within the testicular seminiferous tubules. However, the presence of a circadian clock in the testis is still debatable. For example, a previous study [22] reported a lack of circadian rhythms in testis, whereas another [23] suggested circadian clock gene expression in the testes of multiple species. Hence, investigating rhythmically expressed genes in the testis can shed light on the operating mechanisms of the circadian clocks in the testis.

Circadian clocks have been studied using various model organisms, such as the fruit fly (Drosophila melanogaster) and the zebrafish (*Danio rerio*) [24]. The fruit fly model has been used to understand the generation of circadian clocks, environmental cues for their synchronization, and behavioral patterns, including locomotor activities [25]. The *z*ebrafish model provides a powerful system to investigate light-regulated vertebrate circadian clocks [26]. In particular, the zebrafish is an ideal model organism for studying the roles and mechanisms of the lncRNAs in circadian clocks. The relatively easy accessibility of the early development of zebrafish embryos provides a suitable opportunity for investigating expression profiles and the onset of circadian clocks [24]. The zebrafish pineal gland rhythmically produces melatonin [27], an essential part of the circadian clock system [26]. Zebrafish embryos, cells, tissues, and organs are light-entrainable, which provides unprecedented opportunities for investigating the light input pathway [28]. Moreover, the ability of zebrafish to produce an abundance of offspring, along with the availability of genomic tools, makes zebrafish a suitable vertebrate model for studying the novel lncRNAs and corresponding RNA-seq datasets.

Since RNA-seq datasets address several shortcomings associated with the microarray technology [29], the focus has now been on using RNA sequencing methods to explore RNA expression profiles. For example, a recent study used RNA-seq data to enumerate nearly 8000 human lncRNAs [30]. Despite all of the aforementioned advancement in understanding lncRNAs, there has not been significant progress in predicting the functions of the noncoding RNA genes. In particular, whether or not there is a direct relationship between rhythmically expressed lncRNAs and their lncRNA-encoded peptides remain elusive. Specifically, our understanding of lncRNA-encoded peptides and their implications in circadian regulation requires comprehensive analysis of lncRNA datasets.

In this study, we identified 2507 unannotated testicular transcripts through RNA-seq analysis of the 12-time-point zebrafish testes from two consecutive days with a 4-h interval, and also collected time-series pineal gland lncRNA transcripts and corresponding expression profiles from a published study [31] (Supplementary Figure S1A). We computationally calculated rhythmicity for each of the pineal gland and testis lncRNAs, and predicted top lncRNAs with PCA (principal component analysis). For more than 700 rhythmically expressed lncRNAs, we used their sequences to find the highly similar orthologs in the NCBI database, and exploited the matching sequences to perform GO and KEGG analysis and investigate their regulatory functions. Subsequently, we revealed the overlapping lncRNAs between the zebrafish testis and pineal gland. A conservative analysis was also performed for both testis and pineal gland sequences in order to find their orthologs in humans, mice, and fruit flies. Finally, we predicted conserved, rhythmically expressed, lncRNA-encoded peptides, and computationally predicted their three-dimensional structures and functions. To the best of our knowledge, for the first time, this study provides a comprehensive and coherent comparative analysis of lncRNAs in two important zebrafish organs. The research framework of investigating thousands of lncRNAs with bioinformatic analysis, and its results, will help uncover novel functions of lncRNAs.

## 2. Results

### 2.1. Rhythmically Expressed LncRNAs in the Zebrafish Pineal Gland, and Their GO, COG, and KEGG Analyses

**Figure 1:**
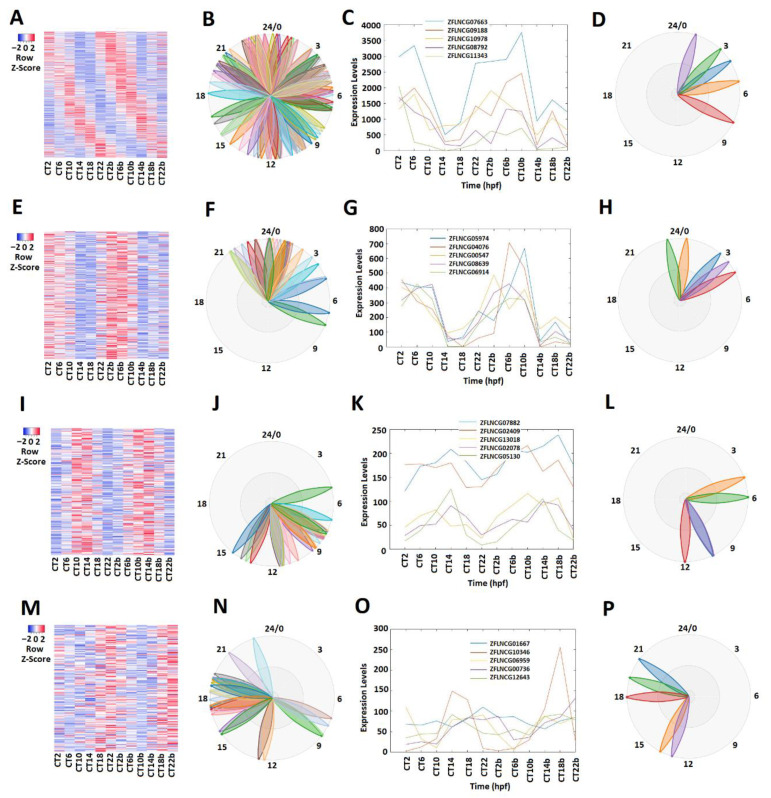
Expression profile analysis of morning (CT 2 and CT 6), evening (CT10 and CT14), and night (CT18 and CT22) rhythmically expressed pineal gland lncRNAs. Analysis of all 586 rhythmically expressed pineal gland lncRNAs (**A**–**D**): heat map (**A**) and phases (**B**) of all 586 rhythmically expressed pineal gland lncRNAs; expression profiles (**C**) and phases (**D**) of representative lncRNAs. Analysis of 241 pineal gland morning lncRNAs (**E**–**H**): heat map (**E**) and phases (**F**) of 241 pineal gland morning lncRNAs; expression profiles (**G**) and phases (**H**) of representative pineal gland morning lncRNAs. Analysis of 196 pineal gland evening lncRNAs (**I**–**L**): heat map (**I**) and phases (**J**) of 189 pineal gland evening lncRNAs; expression profiles (**K**) and phases (**L**) of representative pineal gland evening lncRNAs. Analysis of 149 pineal gland night lncRNAs (**M**–**P**): heat map (**M**) and phases (**N**) of 217 pineal gland night lncRNAs; expression profiles (**O**) and phases (**P**) of representative pineal gland night lncRNAs.

Different circadian clock genes or circadian-clock-controlled genes are expressed in a time-of-day-specific manner, and peak at the specific time of day [32,33,34]. We hypothesized that the rhythmically expressed lncRNAs also display time-of-day-specific expression patterns, as with the circadian clock genes or circadian-clock-controlled genes. In order to find such rhythmic expression patterns, we first investigated the rhythmicity of pineal gland lncRNAs using MetaCycle (Supplementary Table S3). Out of 10,013 lncRNAs, 586 were determined to be rhythmic, with a *p*-value significance level of less than 0.05 (Figure 1A–D; Supplementary Table S5). The phase values of these 586 lncRNAs were calculated and displayed using BioDare2. The heat map (Figure 1A) clearly demonstrated phase correlation among these 586 rhythmic lncRNAs. Next, these rhythmically expressed lncRNAs were further analyzed via principal component analysis (PCA) (Supplementary Figure S2A–D), and the top-ranked rhythmically expressed lncRNAs of the pineal gland (Supplementary Table S6) were selected and their representative phases were plotted as shown in Figure 1B,F,J,N. Furthermore, based on the expression patterns, these rhythmically expressed pineal gland lncRNAs were classified into morning (CT 2 and CT 6), evening (CT 10 and CT 14), and night (CT 18 and CT 22) lncRNAs. Overall, we found 241 morning lncRNAs (E–H), 196 evening lncRNAs (I–L), and 149 night lncRNAs in the zebrafish pineal gland (Supplementary Table S5). The phase (Figure 1C,G,K,O) and period (Figure 1D,H,L,P) diagrams clearly demonstrated the expression patterns for morning lncRNAs, evening lncRNAs, and night lncRNAs, as shown by BioDare2.

These rhythmically expressed pineal gland lncRNAs could be regulated by the circadian clock via E-box, D-box, or RORE. We then interrogated the 5′-promoter sequences of these rhythmically expressed pineal gland lncRNAs, and searched for the presence of E-box, D-box, and RORE elements in their promoter regions (Supplementary Figure S1B). However, due to lack of promoter sequencing information on some lncRNAs, we only found the promoter sequences for 473 rhythmically expressed lncRNAs in the pineal gland, which included the sequences for 190 morning lncRNAs, 159 evening lncRNAs, and 124 night lncRNAs (Supplementary Table S8). We first analyzed the promoter sequences in batch with MATLAB programs to find the direct presence of at least one E-box, one D-box, or one RORE motif (Supplementary Tables S7–S11). The analysis revealed that the 190 morning lncRNAs’ promoter regions each contained the variable motif CANNTG (Supplementary Table S9). However, only 49 evening lncRNAs’ promoter regions contained the D-box motif TTAYGTAA (Supplementary Table S10), while none of the 124 night lncRNAs’ promoter regions contained a RORE motif (Supplementary Table S11), which prompted us to further investigate each of these morning lncRNAs’, evening lncRNAs’, and night lncRNAs’ promoter sequences with the Find Individual Motif Occurrences (FIMO) tool [35]. Interestingly, with a *p*-value threshold of 0.01, we found that 185 morning lncRNAs’, each of the 159 evening lncRNAs’, and 123 night lncRNAs’ promoter sequences, each contained at least one E-Box (Supplementary Table S9), D-box (Supplementary Table S10), and at least one RORE (Supplementary Table S11), respectively. Our promoter sequence analysis suggested that the 190 morning lncRNAs are likely mediated by E-box, the 159 evening lncRNAs by D-box, and the 123 night lncRNAs by RORE (Supplementary Tables S7–S11). Together, these results indicate that most of these rhythmically expressed pineal gland lncRNAs are likely regulated by the circadian clock, and in particular, circadian regulation of morning lncRNAs is likely mediated by E-box, circadian regulation of evening lncRNAs is likely mediated by D-box, and circadian regulation of night lncRNAs is likely mediated by RORE, even though further experimental verification is needed.

**Figure 2:**
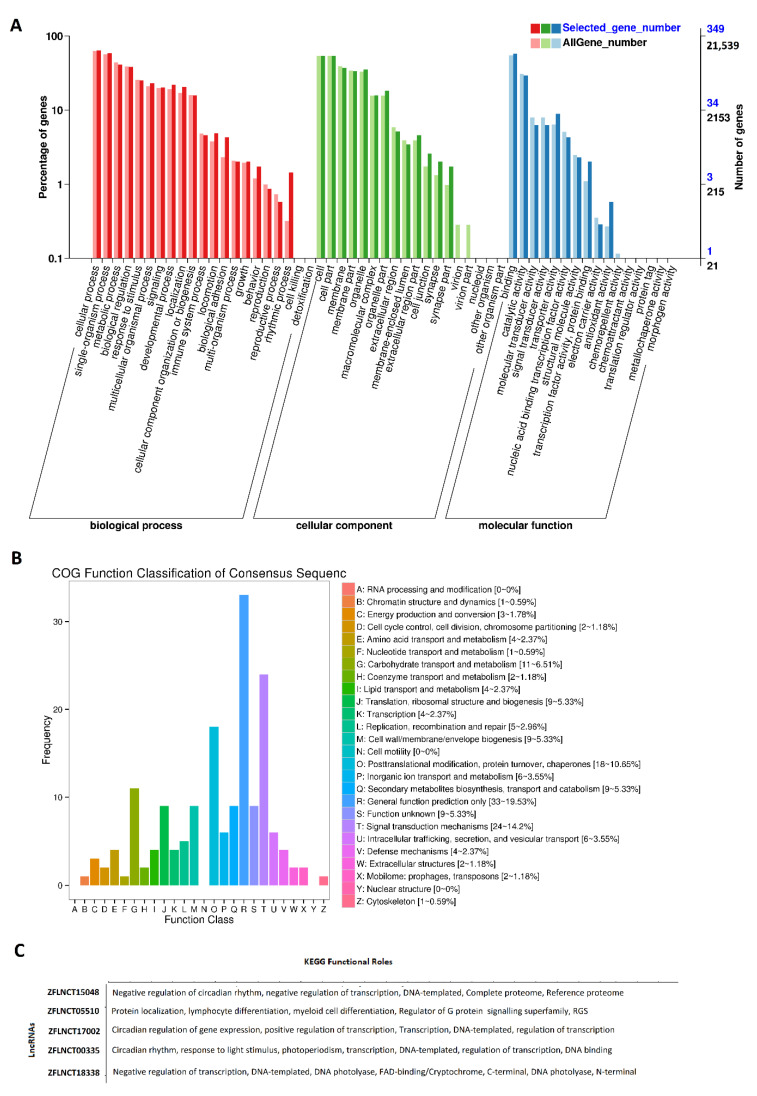
GO, COG, and KEGG analyses of rhythmically expressed pineal gland lncRNAs: GO annotation revealed the percentage of genes involved in different biological processes, cellular components (e.g., cell membrane part, macromolecular complex, and synapse), and molecular functions (**A**), COG functional classification of lncRNAs (**B**), and KEGG functional roles of the representative lncRNAs (**C**).

Gene Ontology (GO) analysis was performed for all of these rhythmically expressed pineal gland lncRNAs (Figure 2A, Supplementary Figures S3–S8, and Supplementary Table S12). The results of the GO analysis (Figure 2A) revealed the percentage of genes involved in diverse sets of biological processes (e.g., cellular process, metabolic process, developmental process, and detoxification), cellular components (e.g., cell membrane part, macromolecular complex, and synapse), and molecular functions (e.g., catalytic activity, transporter activity, and signal transducer activity). The results of the GO analysis (Supplementary Table S12) revealed numerous genomic features for each of these lncRNAs, such as their description, #hits, length, E-value, sim mean, #GO, GO IDs, GO names, enzyme codes, and enzyme names. Out of these rhythmically expressed 586 lncRNAs, over 500 lncRNAs were found to have corresponding names and GO functions in the NCBI database (Supplementary Figures S3 and S4). As expected, several lncRNAs did not have matching sequences and functions in the NCBI; hence, we could not find their GO annotations.

PCA of the GO annotations allowed us to select the top-ranked morning lncRNAs, evening lncRNAs, and night lncRNAs, as shown in Supplementary Figure S5, and more detailed GO interaction diagrams are shown in Supplementary Figures S6–S8. In the GO annotation diagram, each of the nodes represents a unique GO ID and corresponding biological function. As we can see in the diagram (Supplementary Figure S5), an annotation with GO:0003764 represents a molecular function for the given lncRNAs. In doing so, we were able to establish the annotation relationships between different GO IDs for most of the rhythmically expressed zebrafish pineal gland lncRNAs. To the best of our knowledge, this is the first large-scale, comprehensive GO analysis of hundreds of rhythmically expressed pineal gland lncRNAs, providing numerous annotation measures, including enzyme codes and enzyme names.

Subsequently, COG functional classification [36] was performed for all of the pineal gland lncRNAs, and mapped to their Ensembl IDs (Figure 2B). COG classification provided an alternative for efficiently annotating the functional characteristics of novel genomic sequences. The COG annotation of the rhythmically expressed lncRNAs revealed several specific annotations, such as cellular processes and signaling (cell cycle control, cell division, chromosome partitioning), metabolism (amino acid transport and metabolism), and information storage and processing (translation, ribosomal structure and biogenesis).

Furthermore, we performed KEGG pathway analysis on the rhythmically expressed pineal gland lncRNAs mapped to Ensembl gene IDs (Figure 2C, Supplementary Figure S1B, Supplementary Table S13). For each of these lncRNAs, the KEGG pathway analysis revealed their involvement in a diverse set of pathways, such as regulation of the Wnt signaling pathway, or regulation of transcription. Out of the 473 rhythmically expressed lncRNAs with known Ensembl gene IDs, 382 lncRNAs were involved in a variety of biological pathways, such as the metabolic pathway (ZFLNCT08046), and small GTPase-mediated signal transduction (ZFLNCT18335). In particular, ZFLNCT00335 is closely linked to *clockb* (Ensembl gene ID: ENSDARG00000003631), ZFLNCT17002 (Ensembl gene ID: ENSDARG00000011703) is linked to *clocka,* and ZFLNCT18338 is mapped to *cry1bb* (Ensembl gene ID: ENSDARG00000091131). Interestingly, ZFLNCT15048 (Ensembl gene ID: ENSDARG00000075397)—linked to CLOCK-interacting pacemaker a (*cipca*)—and ZFLNCT17588 (Ensembl gene ID: ENSDARG00000078095)—linked to CLOCK-interacting pacemaker b (*cipcb*)—are linked to the negative regulation of circadian rhythm. All of these genes are directly involved in circadian regulation (Supplementary Table S13). Together, we have found 586 rhythmically expressed lncRNAs in the zebrafish pineal gland, and determined their possible involvement in diverse biological processes, including circadian regulation.

### 2.2. Rhythmically Expressed lncRNAs in the Zebrafish Testis, and Their GO, COG, and KEGG Analyses

**Figure 3:**
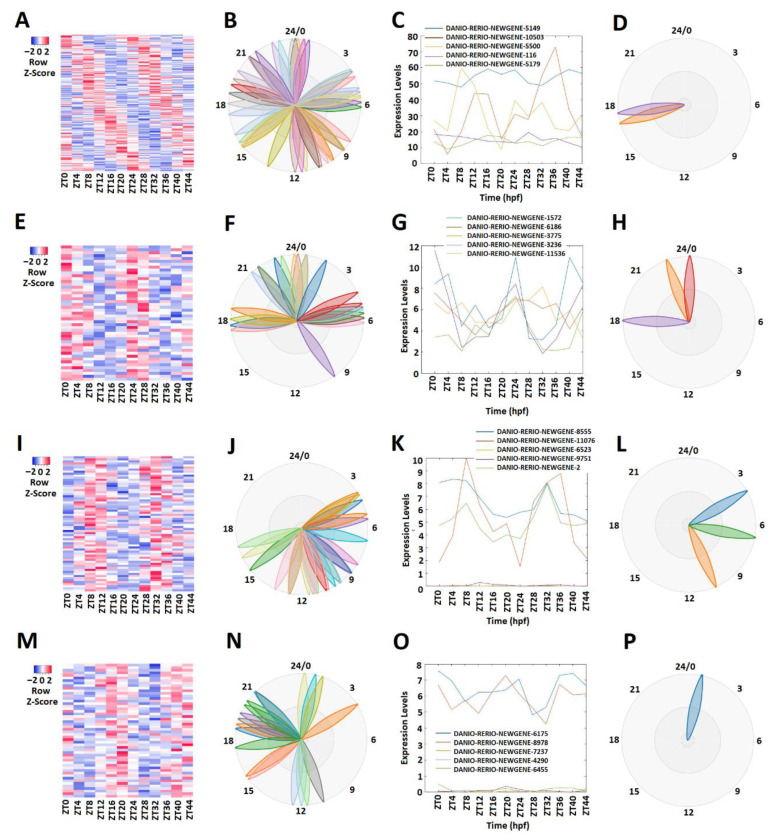
Expression profile analysis of morning (ZT 0 and ZT4), evening (ZT8 and ZT12), and night (ZT 16 and ZT 20) rhythmically expressed testis lncRNAs. Analysis of all 165 rhythmically expressed testis lncRNAs (**A**–**D**): heat map (**A**) and phases (**B**) of all 165 rhythmically expressed testis lncRNAs; expression profiles (**C**) and phases (**D**) of representative lncRNAs. Analysis of 47 testis morning lncRNAs (**E**–**H**): heat map (**E**) and phases (**F**) of 47 testis morning lncRNAs; expression profiles (**G**) and phases (**H**) of representative testis morning lncRNAs. Analysis of 66 testis evening lncRNAs (**I**–**L**): heat map (**I**) and phases (**J**) of 66 testis evening lncRNAs; expression profiles (**K**) and phases (**L**) of representative testis evening lncRNAs. Analysis of 52 testis lncRNAs (**M**–**P**): heat map (**M**) and phases (**N**) of 52 testis night lncRNAs; expression profiles (**O**) and phases (**P**) of representative testis night lncRNAs.

We conducted a similar analysis of the zebrafish testis lncRNAs that we generated in this study. MetaCycle analysis (Supplementary Table S4) determined 165 rhythmically expressed lncRNAs out of 2232 testis lncRNAs (Figure 3A–D; Supplementary Table S14). The rhythmically expressed lncRNAs were further analyzed using expression data. Accordingly, the lncRNAs were classified into morning (ZT0-ZT4) lncRNAs, evening (ZT8-ZT12) lncRNAs, and night (ZT16-ZT20) lncRNAs. Overall, we found 54 morning lncRNAs (Figure 3E–H), 66 evening lncRNAs (I–L), and 52 night lncRNAs (Figure 3M–P) for the zebrafish testis dataset. For each category, we used PCA analysis (Supplementary Figure S3A–D, Supplementary Table S15) to select the representative lncRNAs (Figure 3C,G,K,O). Again, the phase diagrams showed the different expression patterns for morning lncRNAs, evening lncRNAs, and night lncRNAs (Figure 3B,D,F,H,J,L,N,P).

We also interrogated the 5′-promoter regions of these zebrafish testis morning lncRNAs, evening lncRNAs, and night lncRNAs (Supplementary Tables S16–S20). Out of 165 rhythmically expressed testis lncRNAs, we were able to find promoter sequences for 18 lncRNAs, including 8 morning lncRNAs, 8 evening lncRNAs, and 2 night lncRNAs; the sequence analysis using MATLAB revealed the presence of E-box motifs in each of the 8 morning lncRNAs’ promoter sequences (Supplementary Table S18); only 2 of the 8 evening lncRNAs’ sequences contained a D-box motif (Supplementary Table S19). However, none of the night lncRNAs’ sequences contained RORE by direct search (Supplementary Table S20). We further analyzed the morning lncRNAs’, evening lncRNAs’, and night lncRNAs’ promoter sequences with FIMO. Intriguingly, all of the morning, evening, and night sequences were found to contain E-box (Supplementary Table S18), D-box (Supplementary Table S19), and RORE motifs (Supplementary Table S20), respectively. Overall, our promoter sequence analyses of E-box, D-box, and RORE—despite being constrained by the availability of promoter sequencing information—strongly suggest circadian regulation of these rhythmically expressed lncRNAs in the zebrafish testis.

**Figure 4:**
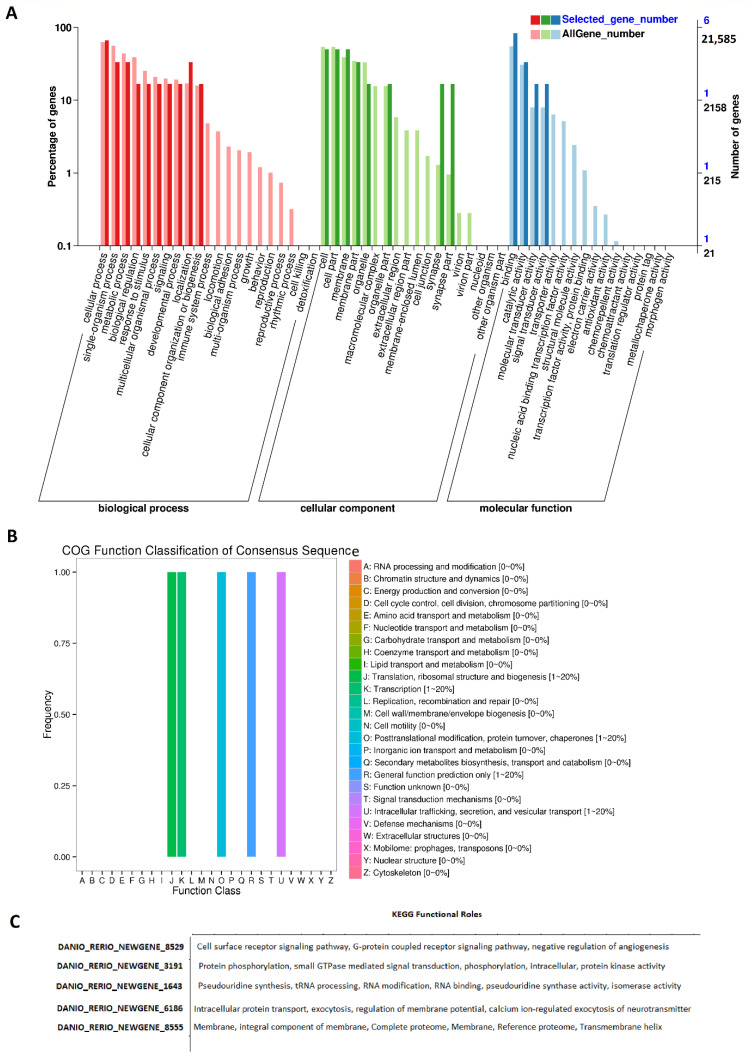
GO, COG, and KEGG analysis of rhythmically expressed testis lncRNAs: GO annotation revealed the percentage of genes involved in different biological processes, cellular components (e.g., cell membrane part, macromolecular complex, and synapse), and molecular functions (**A**), COG functional classification of lncRNAs (**B**), and KEGG functional roles of the representative lncRNAs (**C**).

We also GO annotated all of these rhythmically expressed lncRNAs from the zebrafish testis (Figure 4, Supplementary Figures S10–S14, Supplementary Table S21). The GO analysis revealed the biological functions of more than 143 rhythmically expressed zebrafish testis lncRNAs. The annotation statistics for morning lncRNAs, evening lncRNAs, and night lncRNAs are shown in Supplementary Figure S10. The summary of GO analysis for the morning, evening, and night lncRNAs is shown in Supplementary Figure S11, whereas the detailed GO diagrams are shown in Supplementary Figure S12 (morning lncRNAs), Supplementary Figure S13 (evening lncRNAs), and Supplementary Figure S14 (night lncRNAs). All of the GO annotation measures (GO IDs, GO names, enzyme codes, and enzyme names) for the rhythmically expressed testis lncRNAs are presented in Supplementary Table S21. The GO analysis established various biological functions for most of the rhythmically expressed testis lncRNAs.

We performed COG functional classification for all of these testis lncRNAs mapped to Ensembl IDs (Figure 4B). The COG annotation of the rhythmic lncRNAs revealed several specific annotations, such as information storage and processing (translation, ribosomal structure, and biogenesis), and cellular processes and signaling (post-translational modification, protein turnover, and chaperones).

Furthermore, the KEGG pathway analysis of the rhythmically expressed testis lncRNAs revealed nine lncRNAs active in numerous signaling pathways, such as the cell surface receptor signaling pathway, G-protein-coupled receptor signaling pathway, and negative regulation of angiogenesis (Figure 4C, Supplementary Table S22). Overall, we found 165 rhythmically expressed lncRNAs in the zebrafish testis, and determined their possible involvement in a diverse set of biological processes.

### 2.3. Rhythmically Expressed LncRNAs Shared between the Pineal Gland and the Testis

**Figure 5:**
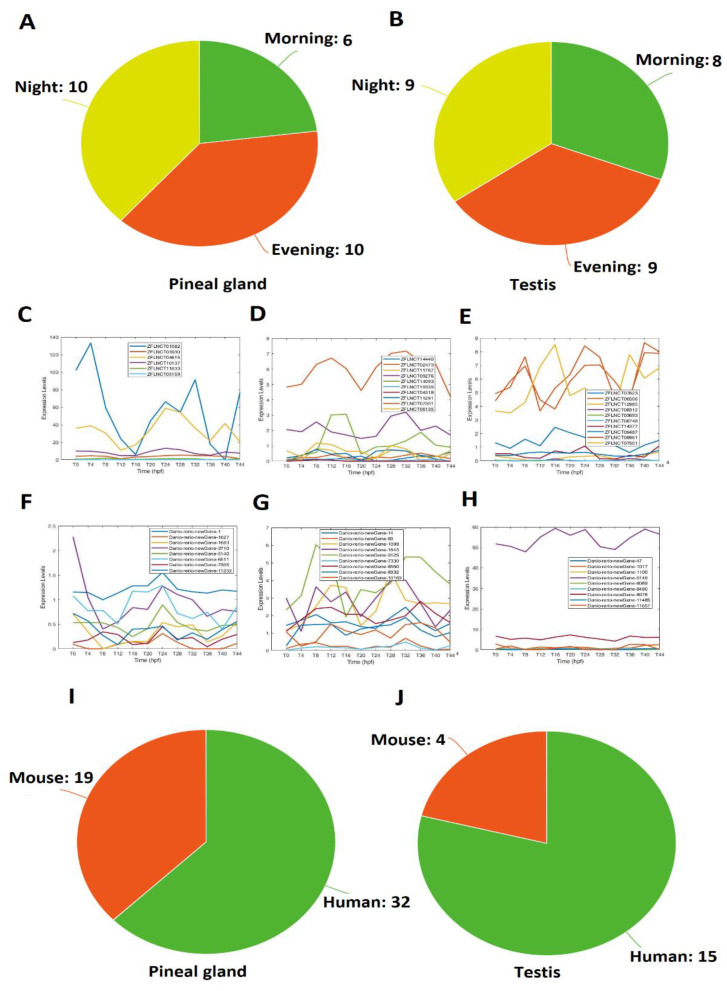
Overlapping lncRNAs between the pineal gland and testis (**A**–**H**), and conserved lncRNAs shared by zebrafish, mice, and humans (**I**,**J**). Fourteen lncRNAs with high similarity between the pineal gland and testis grouped into morning, evening, and night lncRNAs (**A**,**B**). Expression profiles of representative lncRNAs (**C**–**H**): 5 pineal gland morning lncRNAs (**C**), 4 pineal gland evening lncRNAs (**D**), 5 pineal gland night lncRNAs (**E**), 6 testis morning lncRNAs (**F**), 4 testis evening lncRNAs (**G**), and 4 pineal night lncRNAs (**H**). Conserved pineal gland (**I**) and testis (**J**) lncRNAs among zebrafish, mice, and humans.

It is likely that the same lncRNA functions in the two different organs. We investigated the overlapping rhythmically expressed lncRNAs between the zebrafish pineal gland and testis (Figure 5). We performed a local BLAST of the testis lncRNAs against the pineal gland lncRNAs, and revealed that 26 lncRNAs can be found in both the zebrafish testis and the zebrafish pineal gland with a high similarity of E-values lower than E-50 (Supplementary Table S23). We investigated the expression patterns for these 26 lncRNAs and further divided them into morning lncRNAs, evening lncRNAs, and night lncRNAs; based on the testis lncRNA profiles, we identified 8 lncRNAs, 9 lncRNAs, and 9 lncRNAs to be active in morning, evening, and night, respectively. Analysis of pineal gland lncRNA data revealed 6 lncRNAs, 10 lncRNAs, and 10 lncRNAs to be active in morning, evening, and night, respectively. Interestingly, out of the 26 overlapping lncRNAs, we found 6 lncRNAs with similar expression patterns in the testis and the pineal gland. For instance, both the lncRNAs DANIO_RERIO_NEWGENE_14 (testis) and ZFLNCT02473 (pineal gland) were active in the evening (Supplementary Table S24). Together, we have found 26 lncRNAs shared by the zebrafish pineal gland and testis.

### 2.4. Conservation of Zebrafish Rhythmically Expressed LncRNAs with Mice and Humans

It is interesting to determine how many rhythmically expressed pineal and testicular lncRNAs were conserved among humans, mice, and fruit flies. Additionally, we also wanted to estimate whether some of the 14 overlapping lncRNAs between the pineal gland and testis were conserved between these species. An ortholog gene is the homologous gene that is related to those in other organisms by descent from the DNA of a common ancestor [37]. We employed the NCBI BLAST to search for the zebrafish ortholog lncRNAs in humans, mice, and fruit flies. Zebrafish lncRNAs with E-values ≤ 10^−5^ were considered to be orthologs. The BLAST expected value (E-value) suggests the statistical significance threshold for reporting matches of zebrafish lncRNAs against the sequences from these three species. If the statistical significance ascribed to a match was lower than the E-value ≤ 10^−5^ threshold, the matching gene pairs were considered to be potential orthologs.

Out of the 586 rhythmically expressed pineal gland lncRNAs, 32 have human counterparts (Supplementary Table S25). Interestingly, 2 lncRNAs (ZFLNCT11291 and ZFLNCT12985) out of these 32 conserved lncRNAs were part of the 26 overlapping lncRNAs between the pineal gland and testis. The analysis revealed that 19 lncRNAs were conserved with mice, and none of them turned out in these 26 pineal gland/testis overlapping lncRNAs (Supplementary Table S26). We did not find any zebrafish rhythmically expressed pineal gland lncRNAs conserved with fruit flies. Interestingly, 13 pineal gland lncRNAs were conserved among zebrafish, mice, and humans (Supplementary Table S29).

Furthermore, out of 165 rhythmically expressed testis lncRNAs, we found 15 sequences conserved with humans (Supplementary Table S27), out of which 2 lncRNAs (DANIO_RERIO_NEWGENE_1, and DANIO_RERIO_NEWGENE_5125) were part of 14 overlapping lncRNAs between the pineal gland and testis; although 4 testis lncRNAs were conserved with mice (Supplementary Table S28), none of them were part of the overlapping lncRNAs between the pineal gland and testis. Moreover, none of the 165 rhythmically expressed testis lncRNAs were conserved with fruit flies. Interestingly, 3 testis lncRNAs were conserved among zebrafish, mice, and humans (Supplementary Table S29). Taken together, 18 rhythmically expressed lncRNAs were conserved among zebrafish, mice, and humans (Supplementary Table S29). Overall, the conservative analysis suggested that more zebrafish lncRNAs are conserved with humans than with mice.

### 2.5. Conserved LncRNA-Encoded Peptides and Their Predicted 3D Structures

Distinguishing peptides’ coding and noncoding RNAs remains a challenging task, due to lack of information for conserved regions, available protein database, whole-genome sequence, and underlying computational challenges [38,39,40]. Specifically, predicting the 3D structures and functions of the lncRNA-encoded peptides remains elusive. Here, we attempted to determine how many conserved lncRNAs encode peptides, i.e., how many conserved peptides among these three species are encoded by the corresponding conserved lncRNAs.

Interestingly, we found a total of 222 peptides encoded by the 13 conserved zebrafish pineal gland lncRNAs, whereas the corresponding mouse and human orthologs encode 48, and 40 peptides, respectively (Supplementary Tables S30–S32). The conservative analysis of the amino acid sequences revealed that three peptides were conserved among zebrafish, mice, and humans (Supplementary Table S33).

The 3 conserved testicular lncRNAs encode a total of 51 peptides, and the corresponding mouse and human orthologs encode 14 and 13 peptides, respectively (Supplementary Tables S34, S36 and S37). Furthermore, one peptide was conserved among these three species (Supplementary Table S38). Overall, four lncRNA-encoded peptides were conserved among these three species.

**Figure 6:**
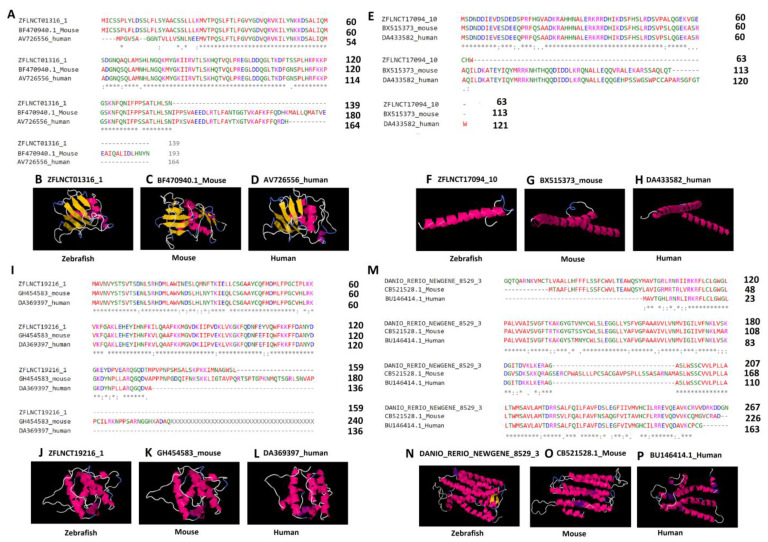
Multiple sequence alignment and three-dimensional structures predicted by (PS)2-v2: protein structure prediction server for the highly conserved lncRNA-encoded peptides among zebrafish, humans, and mice. Pineal gland lncRNA-encoded peptides (**A**–**L**) and testis lncRNA-encoded peptides (**M**–**P**). The asterisk symbols (**A**,**E**,**I**,**M**) depict the identical amino acids among the peptides encoded by zebrafish lncRNAs (ZFLNCT01316_1, ZFLNCT17094_10, ZFLNCT19216_1, and DANIO_RERIO_NEWGENE_8529_1), as well as their corresponding mouse orthologs (BF470940.1_Mouse, BX515373_mouse, GH454583_mouse, and CB521528.1_Mouse) and human orthologs (AV726556_human, DA433582_human, DA369397_human, and BU146414.1_Human). The 3D models of the peptides (**B**–**D**,**F**–**H**,**J**–**L**,**N**–**P**) represent the conservation of α-helix (pink or purple motif structure), β-strand (yellow layered band), and random coils (white or blue thread) among zebrafish, humans, and mice with known domains from the Protein Data Bank, such as, B (2adcA), C (2adcA), D (2adcA), F (1nkpB), G (1nkpB), H (1nkpB), J (1wyoA), K (1wyoA), L (1wyoA), N (1u19A), O (2vt4B), and P (1xioA).

In order to better understand the conserved amino acid sequences, we performed the multiple sequence alignments on lncRNA-encoded peptides. The analysis revealed that a significant portion of peptides shared identical amino acid sequences (Figure 6A,E,I,M). To further evaluate the conservation of peptides, we selected the four conserved lncRNA-encoded peptides for 3D structure prediction, and investigated the three-dimensional structures of the peptides. The amino acid sequences were given as input to the (PS)2-v2: protein structure prediction server for the 3D structure prediction. For each of the six peptides, we tried to predict the 3D structures (see the Methods section, Figure 6). We were able to predict the 3D structures for four peptides from each of the three species, including three pineal gland peptides (ZFLNCT01316_1, ZFLNCT17094_10, and ZFLNCT19216_1) and one testicular peptide (DANIO_RERIO_NEWGENE_8529_1).

Specifically, the 3D models revealed a close similarity among the conserved lncRNA-encoded peptides in terms of α-helix, β-strand, and random coils (Figure 6, Supplementary Figure S15). We further looked into the Protein Data Bank available online: http://www.rcsb.org/ (accessed on 25 May 2021) to find the conserved protein domains for the predicted 3D models (Supplementary Tables S34 and S39).

Interestingly, the zebrafish pineal gland lncRNA-encoded peptide (ZFLNCT01316_1), its mouse ortholog (BF470940.1), and the human ortholog (AV726556) share the known domain 2adcA (Figure 6A–6D) in the Protein Data Bank, whereas the zebrafish lncRNA-encoded peptide (ZFLNCT17094_10) and its mouse ortholog (BX515373) and human ortholog (DA433582) share the known domain 1nkpB (Figure 6E–6H) in the Protein Data Bank. Furthermore, the zebrafish lncRNA-encoded peptide (ZFLNCT19216_1), its mouse ortholog (GH454583), and the human ortholog (DA369397) share the same known domain 1wyoA (Figure 6I–6L) in the Protein Data Bank.

Zebrafish testicular lncRNA-encoded peptide (DANIO_RERIO_NEWGENE_8529_1) was mapped to the known domain 1u19A in the Protein Data Bank, its mouse ortholog (CB521528.1_Mouse) was mapped to the known domain 1xioA in the Protein Data Bank, and the human ortholog (BU146414.1_Human) was mapped to the known domain 2vt4B in the Protein Data Bank (Supplementary Table S39). Despite mapping to different known domains, the testicular lncRNA-encoded peptides showed a close resemblance of α-helix, and random coils in the 3D models (Figure 6M–P).

Together, we found four peptides encoded by rhythmically expressed zebrafish lncRNAs from the pineal gland and testis, shared by the three species. The predicted 3D structures will help us to better understand the novel functional mechanisms of lncRNAs in the zebrafish pineal gland and testis.

## 3. Discussion

It is increasingly evident that lncRNAs are an important regulator of numerous fundamental biological processes [5,6]. Although the number of lncRNAs and corresponding expression profiles is continuously increasing [3,4,31], their roles in circadian regulation remain elusive. In particular, only a few circadian-clock-regulated lncRNAs have been investigated thus far [16,17]. In particular, comparison of rhythmically expressed lncRNAs in different tissues/organs of zebrafish has not been conducted before. Our study provides numerous interesting insights for the lncRNAs involved in circadian clock research. For example, while a previous study [19] identified rhythmic expression patterns in 112 lncRNAs in the rat pineal gland, and studied 8 lncRNAs for circadian oscillations in the suprachiasmatic nucleus, our study revealed over 700 rhythmically expressed lncRNAs in zebrafish. In this study, for the first time, we compared rhythmically expressed lncRNAs between the pineal gland and the testis. Based on the expression profiles, the rhythmically expressed lncRNAs were classified into morning, evening, and night groups. We reasoned that morning, evening, and night lncRNAs should be regulated by the circadian clock via the E-box, D-box, or RORE, respectively [29,30,31]. As expected, our comprehensive promoter analysis supported the hypothesis that the morning, evening, and night lncRNAs were likely regulated by the E-box, D-box, and RORE elements, respectively. We also found 14 lncRNAs shared by the pineal gland and the testis. The cross-species conservative analysis revealed that a number of zebrafish rhythmically expressed pineal gland and testis lncRNAs shared close similarity in those of both humans and mice, but none of them appeared to be closely related to any in fruit flies. Furthermore, several lncRNAs harbor sORFs that carry the potential to encode peptides [14,15]. We found a combined 18 rhythmically expressed lncRNAs in the pineal gland and testis, encoding hundreds of peptides, including six lncRNA-encoded micropeptides conserved among zebrafish, mice, and humans. Hence, we computationally predicted the 3D structures of these six peptides. In particular, 3D models of five out of six peptides show the highly conserved α-helix, β-strand, and random coils with known domains in the Protein Data Bank.

Despite the promising findings, our study entails certain bioinformatic limitations. For example, we employed RNA-seq technology to characterize and quantify the RNA transcripts in order to investigate the pineal gland and testis lncRNAs. However, the RNA-seq methods come with numerous limitations [41], such as employing the poly(A) tails to predict genomic sequences [42]. This approach tends to miss out some of the novel genomic sequences, including the low-abundance transcripts. Moreover, our bioinformatic predictions—such as promoter regulation by E-Box, D-Box, and RORE regulatory elements—require additional experimental validation. Ribosome profiling can be employed to experimentally verify the computationally predicted lncRNA-encoded peptides. In particular, our study could only find conserved lncRNA-encoded peptides containing < 150 amino acids. These peptides are technically not micropeptides, because the latter are arbitrarily defined as the small peptides containing < 100 amino acids [11]. However, our framework can also be equally applied to investigate small micropeptides and their conservation in different species. Furthermore, due to the lack of the available zebrafish tissue-/organ-specific gene expression profiles, we compared the expression patterns measured using different treatments and different time points. As such, the zebrafish pineal gland data were profiled under constant darkness, whereas the zebrafish testis data were profiled under LD conditions. Nevertheless, we were able to reveal 165 rhythmically expressed lncRNAs in the testis—which shall shed light on the testicular rhythmicity—and, particularly, 14 lncRNAs shared by the pineal gland and the testis. However, owing to the tissue- and light-treatment-specific variations, the common and conserved lncRNAs and expression profiles will require further experimental validation.

Our findings will expedite the ongoing research of the circadian system in zebrafish by combining experimental observations and computational mechanisms. The proposed computational framework will potentially help to develop novel techniques for curing lncRNA-regulated and dysrhythmia-derived human diseases. We believe that, as more data and computational tools become available in the future, our integrative framework that combines experimental responses and computational expertise can be used to further shed light on the crucial roles of noncoding RNAs in specific human diseases.

## 4. Materials and Methods

### 4.1. Zebrafish Rhythmically Expressed RNA-Seq Datasets for the Pineal Gland and Testis

We conducted transcriptome analysis of adult testes in a time-series manner, i.e., each with two duplicates collected for consecutive two days with a 4-h interval under LD conditions, each sample with mixed testes from three adult fish. RNA-seq-based transcriptome analysis was conducted as described previously [43]. Total RNAs from each sample were extracted with TRIzol (Invitrogen). The RNA sequencing of these total testicular RNAs was performed as follows. *1. Library construction for sequencing*. A total amount of 3 μg RNA per sample was used for constructing sequencing libraries, which were generated using NEBNext^®^Ultra™ RNA Library Prep Kit (NEB, USA), following the manufacturer’s instructions. The library preparation was not strand-specific. *2. Clustering and sequencing*. Clustering of the index-coded samples was performed on a cBot Cluster Generation System using TruSeq PE Cluster Kit (Illumina, PE-401-3001), according to the manufacturer’s instructions. After clustering, the library preparations were sequenced on an Illumina HiSeq X 10 platform. *3. Quality control*. We calculated the Q20, Q30, GC content, and duplication data and then generated the raw reads. All of the following analyses were based on clean data with high quality. *4. Transcriptome assembly*. Transcriptome assembly was performed as below; these clean reads were mapped to the zebrafish genome (GRCz 11) with the standard hisat2 command template implemented in Hisat2 tool software [44]. Only reads with a perfect match or one mismatch were further analyzed and annotated based on the reference genome. Overall, we generated 2507 novel transcripts from our transcriptome analysis of the 3-month-old wild-type (WT) zebrafish testis. The data include 12 time points with 4-h intervals for a consecutive 48 h—ZT0, ZT4, ZT8, ZT12, ZT16, ZT20, ZT24, ZT28, ZT32, ZT36, ZT40, and ZT44—and each time point with duplicates (Supplementary Table S2). The Zeitgeber time (ZT) represents the standard 24-h duration of the entrained cycle under LD (14-h L/10-h D) condition, with ZT0 and ZT12 representing the start and end of the day, respectively [45]. The testis expression profiling data are available in Supplementary Table S2, and in the NCBI Sequence Read Archive (SRA) database, with accession number PRJNA579855. The pineal gland RNA-Seq circadian data for adult transgenic zebrafish (0.5–1.5 years old), *Tg(aanat2:EGFP)Y8*, expressing EGFP, were generated from a previous study [31] and taken from the online database. The circadian data included 12 time points with 4-h intervals for two consecutive days—CT14, CT18, CT22, CT2, CT6, CT10, CT14b, CT18b, CT22b, CT2b, CT6b, and CT10b (Supplementary Table S1). The circadian time (CT) indicates the subjective time of an organism in the circadian cycle under constant darkness (DD), with CT0 and CT12 representing the beginning of a subjective day and night, respectively [45].

### 4.2. Collection of Zebrafish Pineal Gland LncRNAs

A total of 21,128 lncRNA transcripts were selected from the ZFLNC database [46]. These lncRNAs were assembled from Ensembl, NCBI, NONCODE available online: www.noncode.org (accessed on 20 May 2021), and zflncRNApedia available online: http://genome.igib.res.in/zflncRNApedia/about.html (accessed on 18 May 2021). Overall, the ZFLNC database catalogues the largest collection of zebrafish lncRNAs to date. Out of the whole dataset, 10,013 lncRNAs were expressed in the pineal gland for at least one time point and, hence, they were selected for our analysis.

### 4.3. Identification of Zebrafish Testis LncRNAs

We hypothesize that some of the 2507 novel testis transcripts generated in this study may be lncRNAs. To identify lncRNAs from these zebrafish testis transcripts, we performed a local BLAST of 2507 testis transcripts against the ZFLNC lncRNAs [46], and determined that 1830 testis transcripts were lncRNAs, as they showed high similarity in expected value (E-value) lower than E-50 with ZFLNC lncRNAs. The remaining 677 testis transcripts did not show E-value-based similarity with ZFLNC lncRNAs. However, as the ZFLNC lncRNA transcripts were far from complete, we assumed that some of these 677 testis transcripts might still be classified as lncRNAs. There are numerous computational tools that can be applied to investigate the coding potential of the novel lncRNAs [47]. However, the Coding-Potential Assessment Tool (CPAT) [48] has the best sensitivity and specificity of 0.96 and 0.97, respectively. Hence, we computed the coding probabilities of these 677 transcripts using the logistic regression model implemented with CPAT [48]. We then identified an additional 402 transcripts as lncRNAs based on their having been predicted to have coding probabilities less than 0.1. Together, we have a total of 2232 lncRNAs identified from the 2507 novel transcripts from zebrafish testis.

### 4.4. Rhythmicity Analysis

Identifying periodic signals in the time-course-based RNA-seq data is important in studying oscillatory systems, such as the circadian clock [49]. In this study, we investigated the rhythmicity of circadian RNA-seq datasets using MetaCycle [50], an R language-based software suite that combines algorithms implemented with ARSER, JTK_CYCLE, and Lomb–Scargle to estimate rhythmicity in time-series data. MetaCycle has two core functions—*meta2d* and *meta3d*—designed to analyze two-dimensional and three-dimensional time-series datasets, respectively. MetaCycle provides an integrated *p*-value for the time-series data. LncRNAs with a *p*-value less than 0.05 were considered to be rhythmic. Out of 10,013 pineal gland lncRNAs, 586 were determined to be rhythmic, with a *p*-value significance level less than 0.05, while out of the 2232 testis lncRNAs, only 165 were rhythmically expressed, with less than 0.05 *p*-values. These rhythmically expressed lncRNAs were selected for further investigation. Since the pineal gland and testis time-series datasets were profiled on differing time points, we used BioDare2 systems available online: https://biodare2.ed.ac.uk/ (accessed on 15 May 2021) [51] for comparative visualizations of the phase and period analysis of the corresponding rhythmic lncRNAs.

### 4.5. Searching for E-Box, D-Box, and RORE Regulatory Motifs in the Promoter Regions of the Rhythmically Expressed LncRNAs

We assumed that the rhythmically expressed lncRNAs could be regulated by the circadian clock via E-Box, D-box, or RORE. We selected 5000 base pairs of 5’ upstream promoter nucleotide sequences of the lncRNA orthologs genes from Ensembl, identified using BLAST. Subsequently, we searched for the presence of E-Box, D-Box, and RORE regulatory elements for morning lncRNAs, evening lncRNAs, and night lncRNAs, respectively [32,33,34]. The E-Box contains a variable motif CANNTG, D-Box contains the motif TTAYGTAA (where Y is pyrimidine T or C nucleotide), and ROR response elements (RORE) contain the variable motif (A/T)A(A/T)NT(A/G)GGTCA (where N can be any nucleotide). The promoter sequences for these lncRNAs were selected after NCBI BLAST with Blast2GO. BLAST output provided GenBank accession numbers, which were further mapped to Ensembl gene IDs using bioDBnet (biological DataBase network) [52] application (Supplementary Figure S1B, Supplementary Tables S8 and S17). For each of the Ensembl gene IDs, 5000 nucleotides’ long 5’ upstream promoter sequences were downloaded from Ensembl BioMart. The presence of E- Box, D- Box, and RORE in the promoter sequences was analyzed in batches using both the Find Individual Motif Occurrences (FIMO) motif search tool [35] and MATLAB (The Mathworks, Inc.) software. The E-Box, D-box, or RORE motif probability distributions for FIMO were taken from the JASPAR [53] online application. The FIMO match *p*-value threshold was kept to 0.01.

### 4.6. GO Analysis, COG Analysis, and KEGG Pathway Analysis of Rhythmically Expressed LncRNAs

GO analysis is a major bioinformatics initiative to unify the representation of gene and gene product attributes across all species [54]. To investigate and understand the functional enrichment of the rhythmically expressed zebrafish pineal gland and testis lncRNAs, we performed Gene Ontology (GO) [55] analysis of all rhythmically expressed lncRNAs. The functional information of lncRNAs was represented via GO annotation—a controlled vocabulary of functional attributes. We exploited the BLAST [56] algorithm integrated with Blast2GO [57], a bioinformatics software suit for automatic, high-throughput functional annotation of novel lncRNA sequences. Blast2GO makes use of the BLAST algorithm to identify similar sequences, and then it transfers existing functional annotation from the characterized sequences to the novel one. For all of these zebrafish lncRNAs, neither the gene names nor transcript IDs were available. Therefore, in order to predict the gene names, functions, etc. for the given sequences, we applied the BLAST algorithm to BLAST all of the zebrafish sequences against the NCBI’s nucleotide collection (nt) database, in order to obtain the desirable ortholog sequences. In order to understand the functional annotations of the lncRNAs, we investigated their orthologs. Furthermore, for all of the lncRNAs mapped to Ensembl gene IDs, we applied BMKCloud available online: https://international.biocloud.net/ (accessed on 15 May 2021) tools for Clusters of Orthologous Groups (COG) [36] analysis and visualization of GO analysis. The BMKCloud GO analysis revealed their roles in different biological processes, cellular components, and molecular functions. COG analysis was applied for functional classifications (such as RNA processing and modification, chromatin structure and dynamics, cell cycle control, and mitosis) of the genes from newly sequenced genomes.

We further performed Kyoto Encyclopedia of Genes and Genomes (KEGG) pathway analysis in order to investigate the involvement of the rhythmically expressed testis and pineal gland lncRNAs in different signaling pathways. The manually drawn KEGG pathway mapping was performed using the Database for Annotation, Visualization, and Integrated Discovery (DAVID) v6.8 [58].

### 4.7. Principal Component Analysis (PCA)

PCA is a powerful statistical technique to compress multiple dimensions of a dataset into a few dimensions, where each dimension is termed as a principal component [59]. We applied PCA analysis to separately rank the morning, evening, and night lncRNAs based on their expression profiles from the zebrafish pineal gland and testis expression datasets. The PCA analysis compressed the multidimensional datasets into multiple principal components, and assigned the lncRNAs different scores in each of the principal components. We ranked the lncRNAs based on the absolute scores in the first principal component, and plotted the top-ranked representative lncRNAs with high PCA scores from both the datasets.

### 4.8. Conservation Analysis with NCBI BLAST

The study exploited the NCBI BLAST algorithm for comparing genomic sequences. The BLAST expected value (E-value) was used to assess the sequence similarity. E-value essentially represents the statistical probability of the number of alignments that are expected to occur by chance, in a database of a given size, with a score greater than or equal to the BLAST raw score. A low BLAST E-value is indicative of high statistical significance. If the BLAST expected value was higher than the threshold E-value, the match was not reported.

### 4.9. Prediction of lncRNA-Encoded Peptides’ 3D Structure and Functions

In this study, for each of the conserved lncRNAs, we applied ExPASy [60] to calculate the amino acid sequences for all possible peptides. Subsequently, for each of the zebrafish lncRNA-encoded peptides, we separately performed multiple sequence alignment with the peptides encoded by the lncRNAs’ human and mouse orthologs. The multiple sequence alignment among the peptides from these three species was performed using Clustal Omega available online https://www.ebi.ac.uk/Tools/msa/clustalo/ (accessed on 15 May 2021). The three-dimensional structure and functions of the conserved peptides were predicted using (PS)2-v2: protein structure prediction server [61,62]. All of the 3D structures were visualized using Jmol available online http://www.jmol.org/ (accessed on 15 May 2021)—an open-source Java viewer of 3D structures of biomolecules.

## 5. Conclusions

We identified more than 700 rhythmically expressed lncRNAs, and examined their KEGG, GO, and COG functions in the zebrafish pineal gland and testis. The 14 overlapping lncRNAs between the pineal gland and testis provide a unique insight into understanding the relationships between the functions of two different zebrafish organs. The conservative analysis helped find some zebrafish lncRNA orthologs in humans and mice. Furthermore, investigating lncRNA-encoded peptides and corresponding 3D structures enables a comprehensive understanding of the novel lncRNAs. Together, integration of experimental data and computational biology to identify lncRNAs will help to uncover corresponding encoded peptides and reveal their functions in circadian regulation.

## Supplementary Materials

The following are available online at https://www.mdpi.com/article/10.3390/ijms22157810/s1.
