# Supplementary figures and images for: Identification of Rhythmically Expressed LncRNAs in the Zebrafish Pineal Gland and Testis
Source: Int J Mol Sci. 2021 Jul 22;22(15):7810. doi: 10.3390/ijms22157810 (PMC8346003; doi:10.3390/ijms22157810)

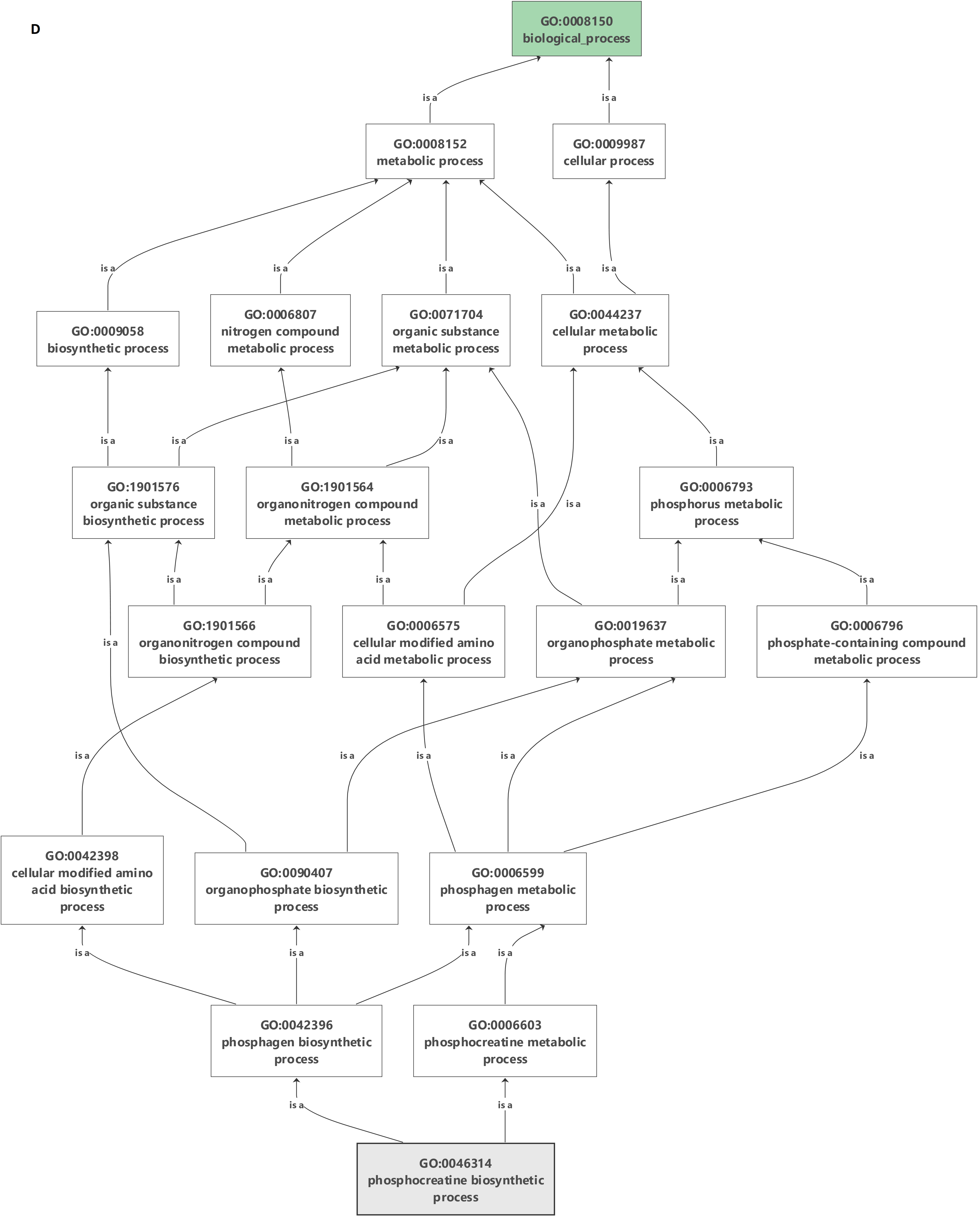

Supplement: Supplementary file 1 [file ijms-22-07810-s001.zip › SupplementaryTables&Figures/Supplementary_Figures/SupplementaryFigure 7.tiff]

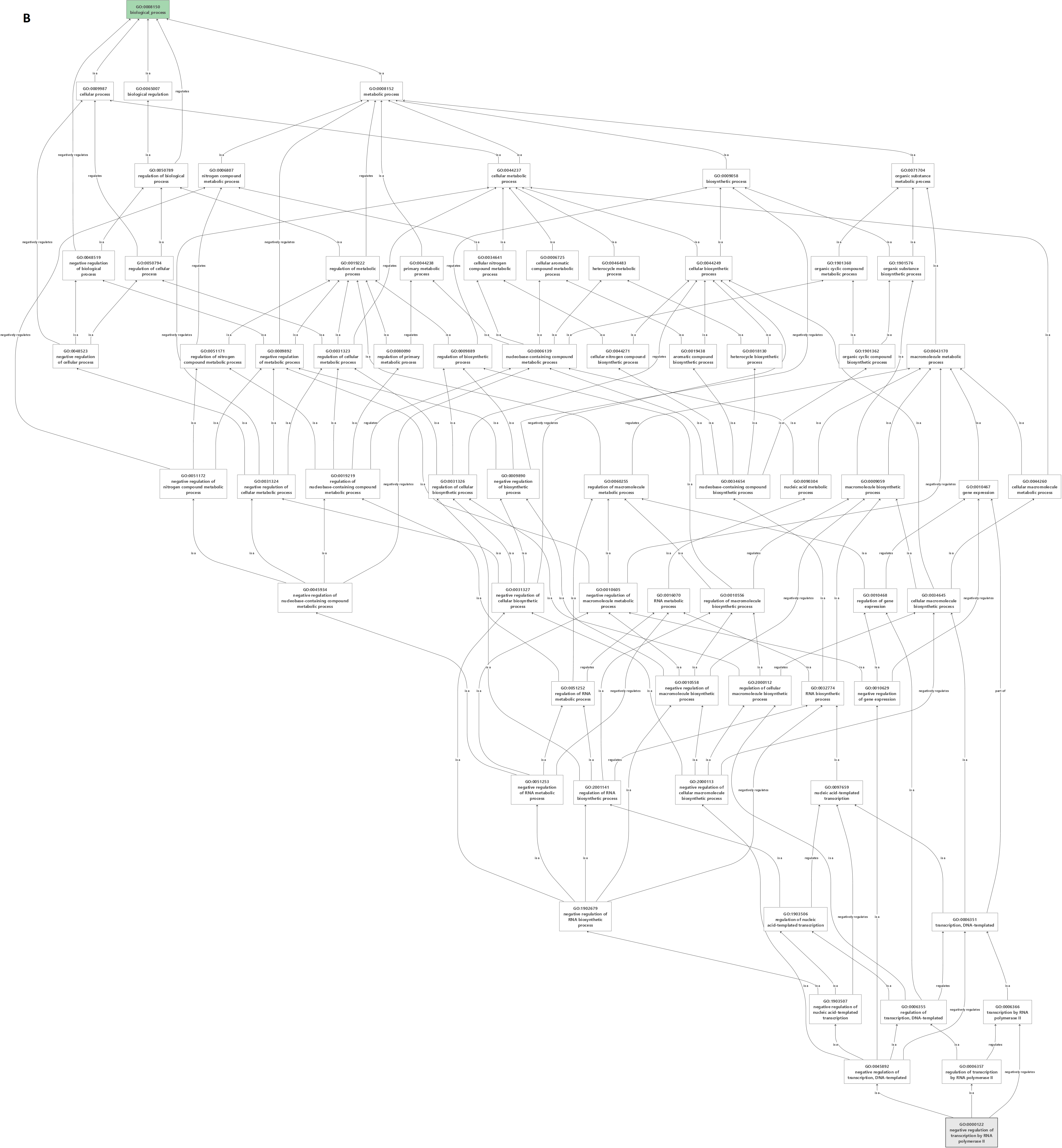

Supplement: Supplementary file 1 [file ijms-22-07810-s001.zip › SupplementaryTables&Figures/Supplementary_Figures/SupplementaryFigure 12.tiff]

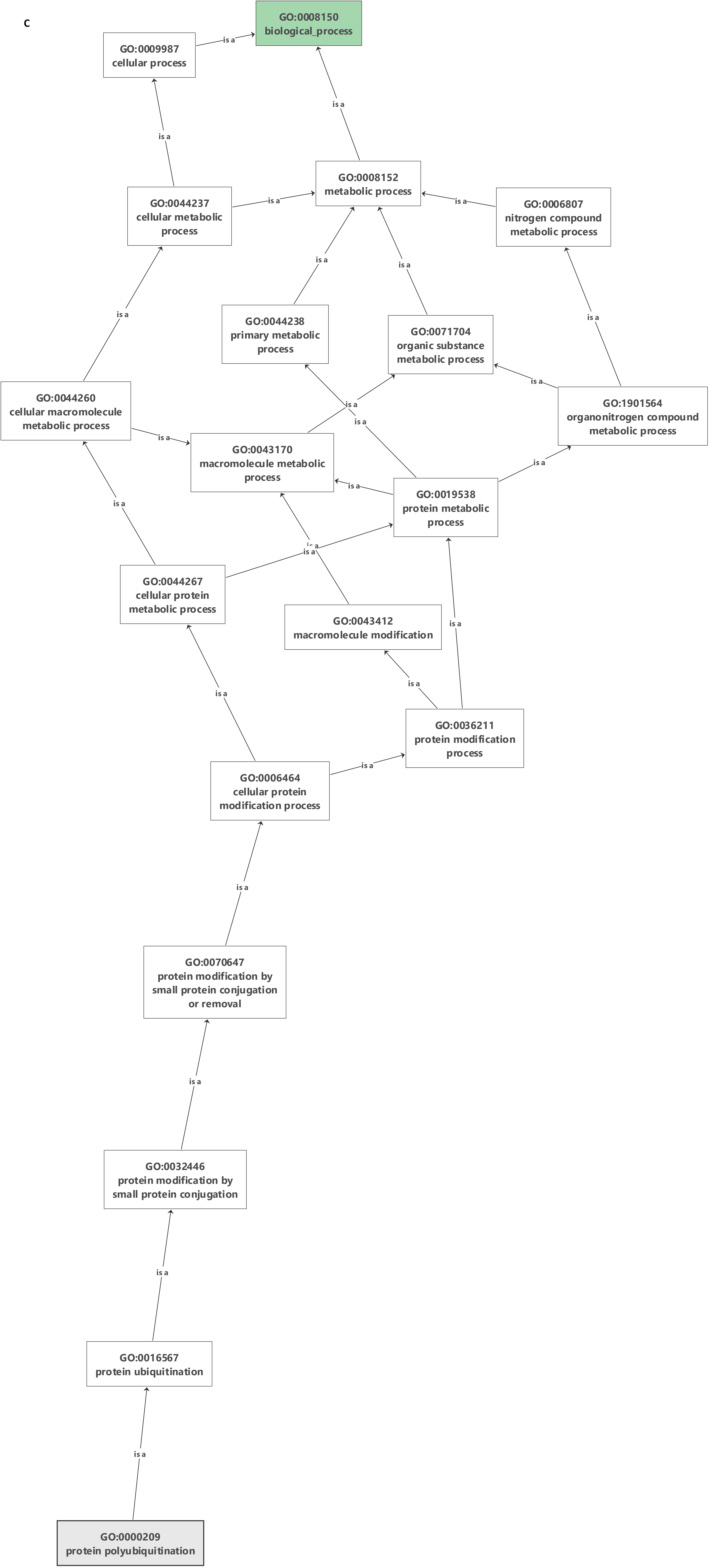

Supplement: Supplementary file 1 [file ijms-22-07810-s001.zip › SupplementaryTables&Figures/Supplementary_Figures/SupplementaryFigure 13.tiff]

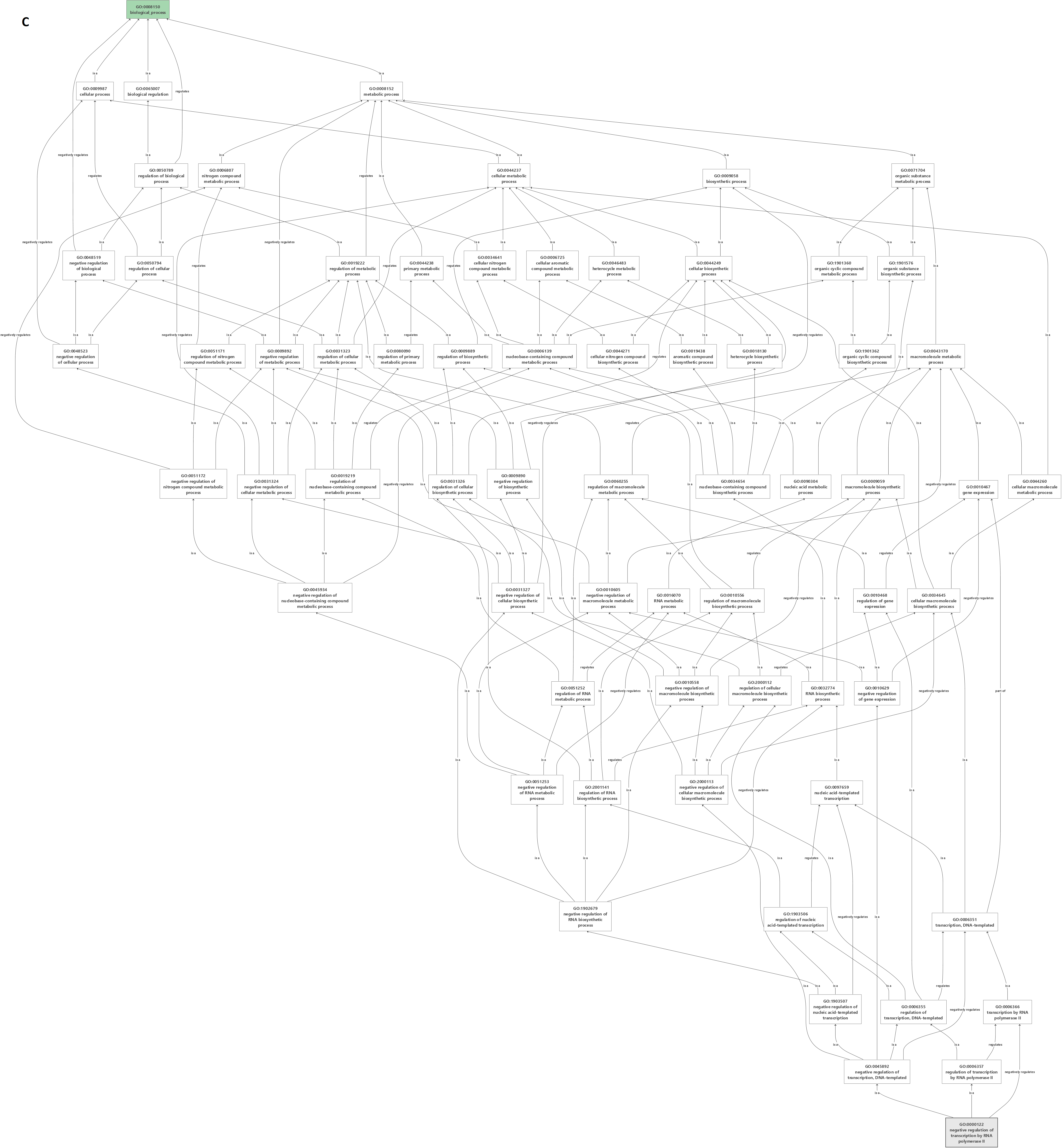

Supplement: Supplementary file 1 [file ijms-22-07810-s001.zip › SupplementaryTables&Figures/Supplementary_Figures/SupplementaryFigure 6.tiff]

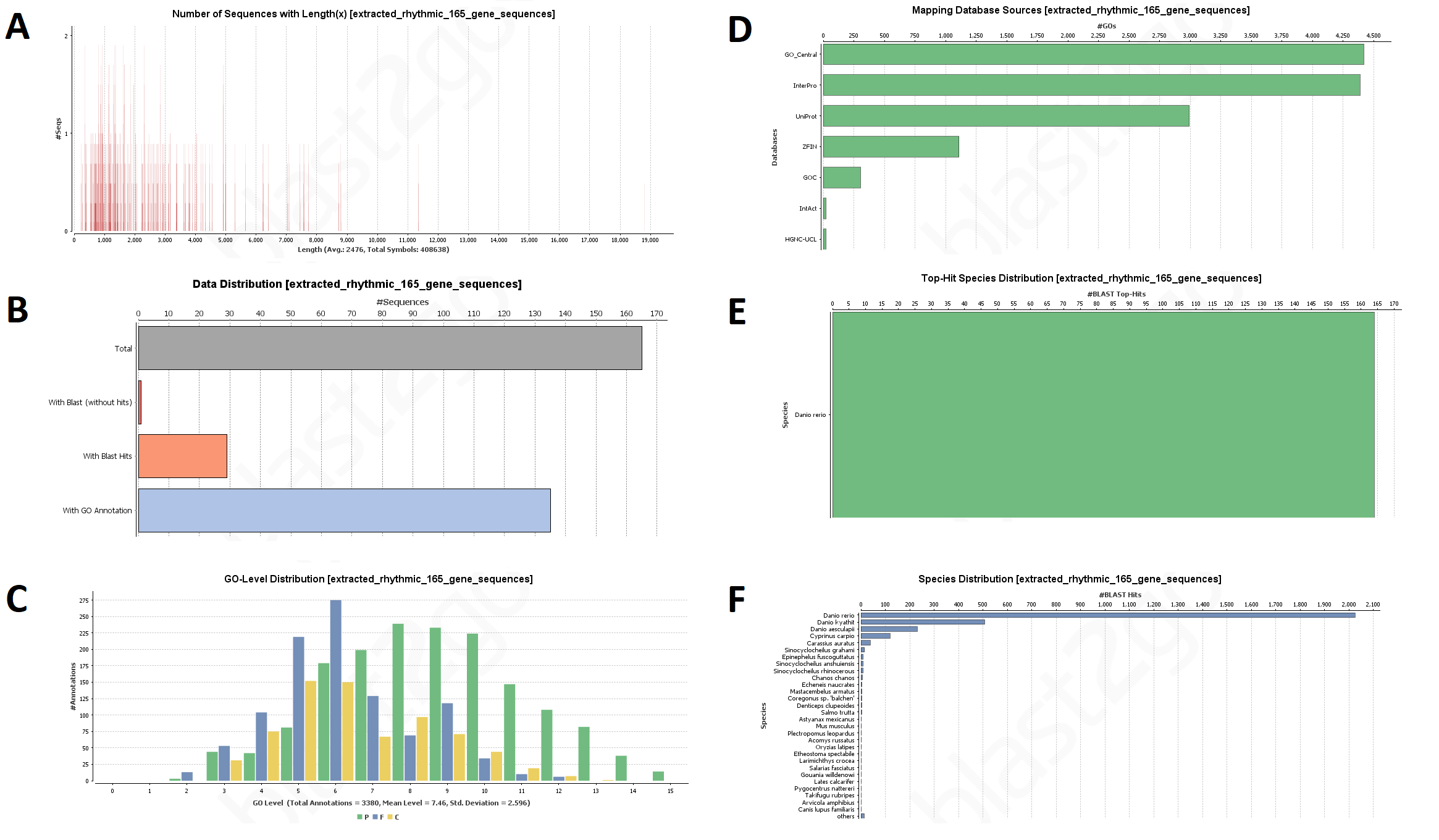

Supplement: Supplementary file 1 [file ijms-22-07810-s001.zip › SupplementaryTables&Figures/Supplementary_Figures/SupplementaryFigure 10.tif]

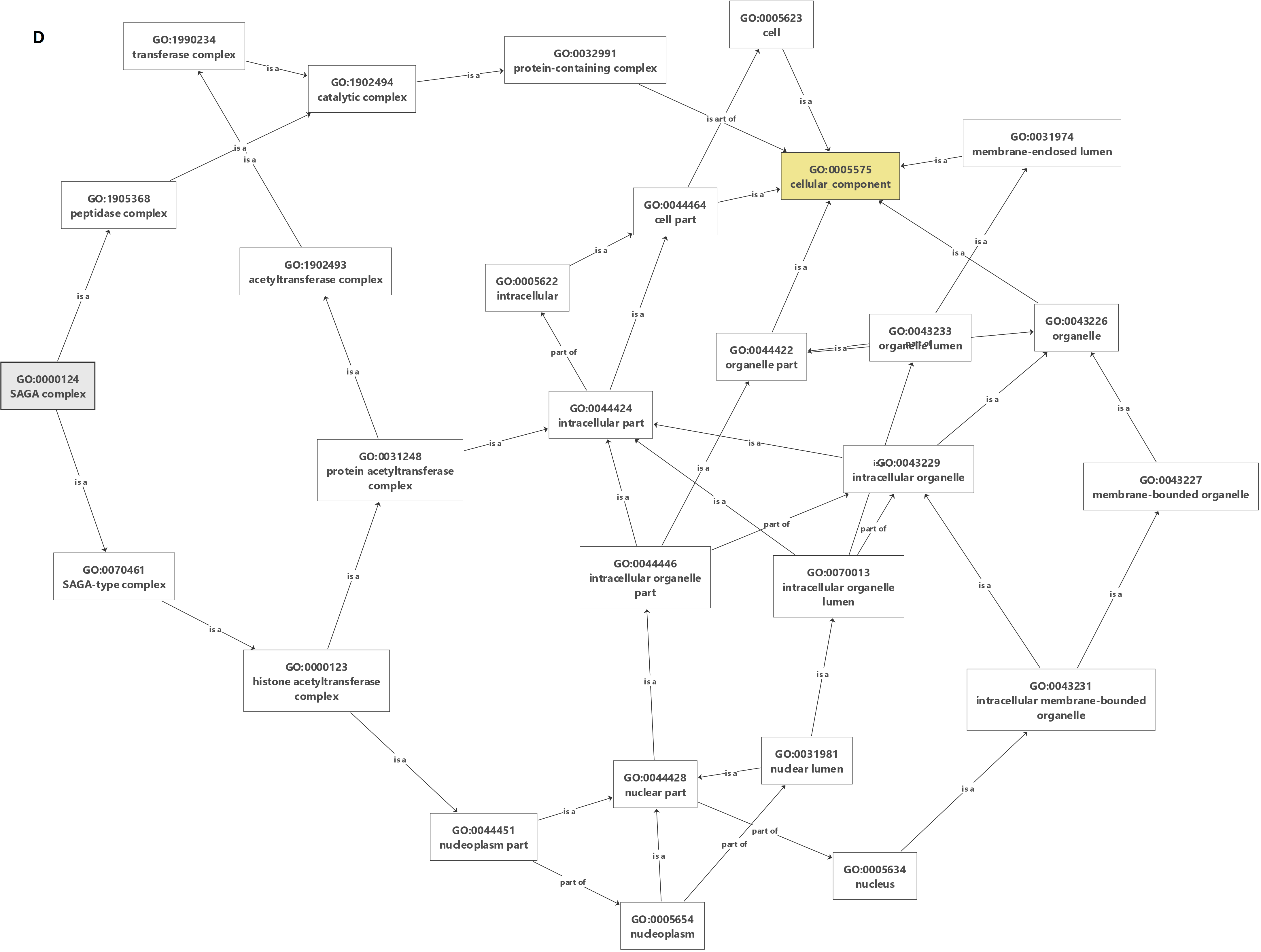

Supplement: Supplementary file 1 [file ijms-22-07810-s001.zip › SupplementaryTables&Figures/Supplementary_Figures/SupplementaryFigure 14.tiff]

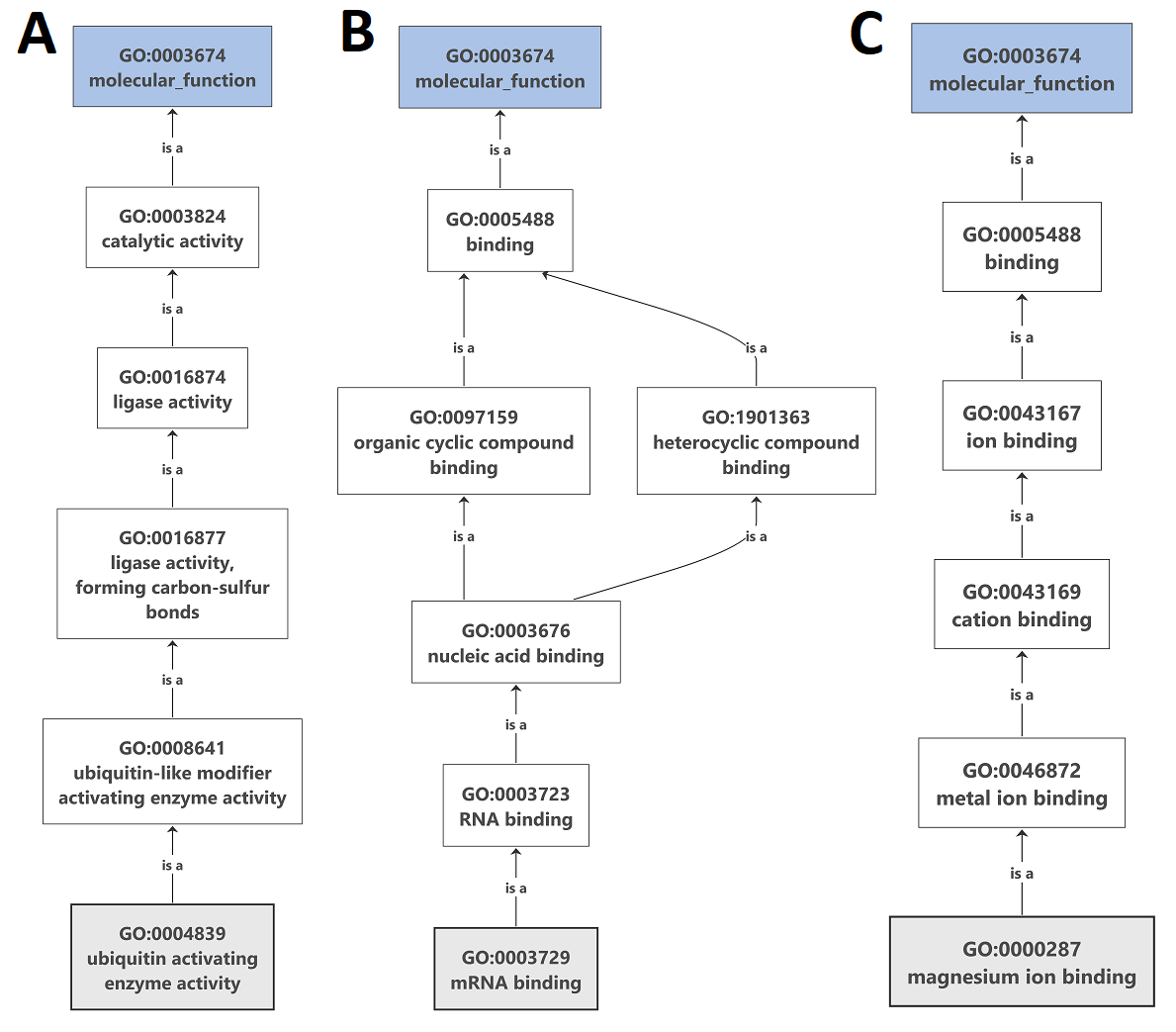

Supplement: Supplementary file 1 [file ijms-22-07810-s001.zip › SupplementaryTables&Figures/Supplementary_Figures/SupplementaryFigure 11.tif]

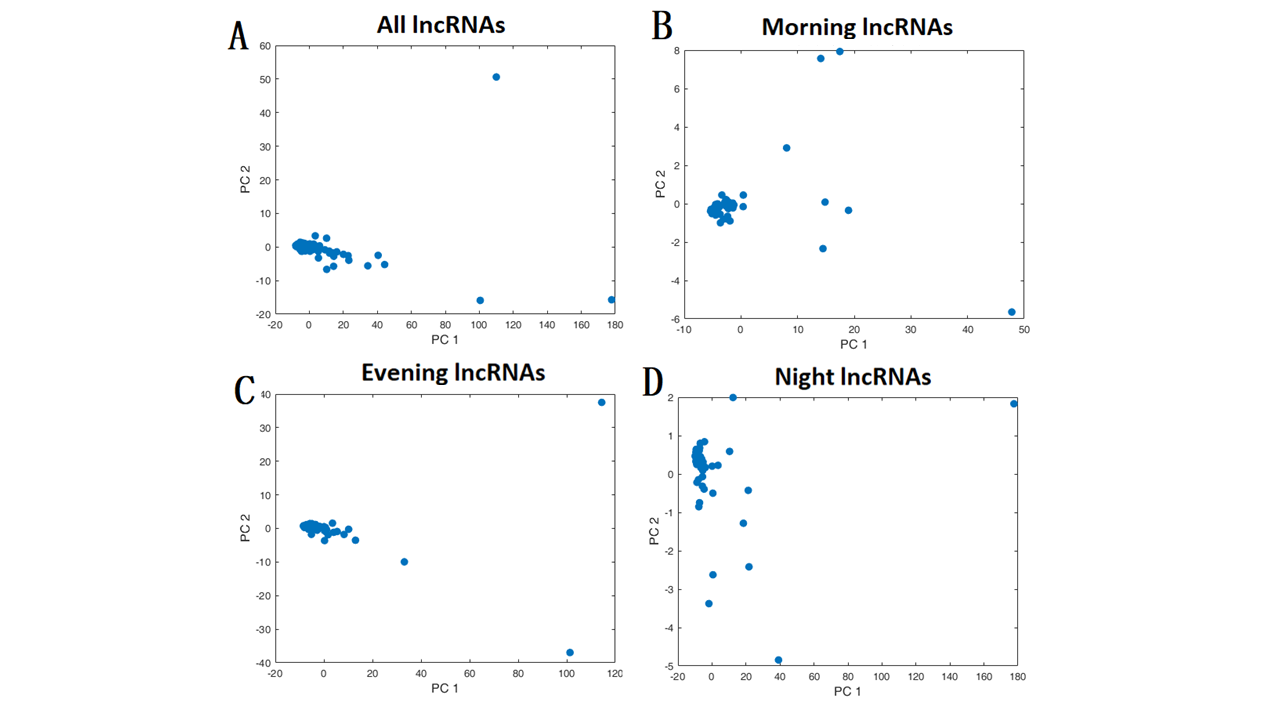

Supplement: Supplementary file 1 [file ijms-22-07810-s001.zip › SupplementaryTables&Figures/Supplementary_Figures/SupplementaryFigure 9.tif]

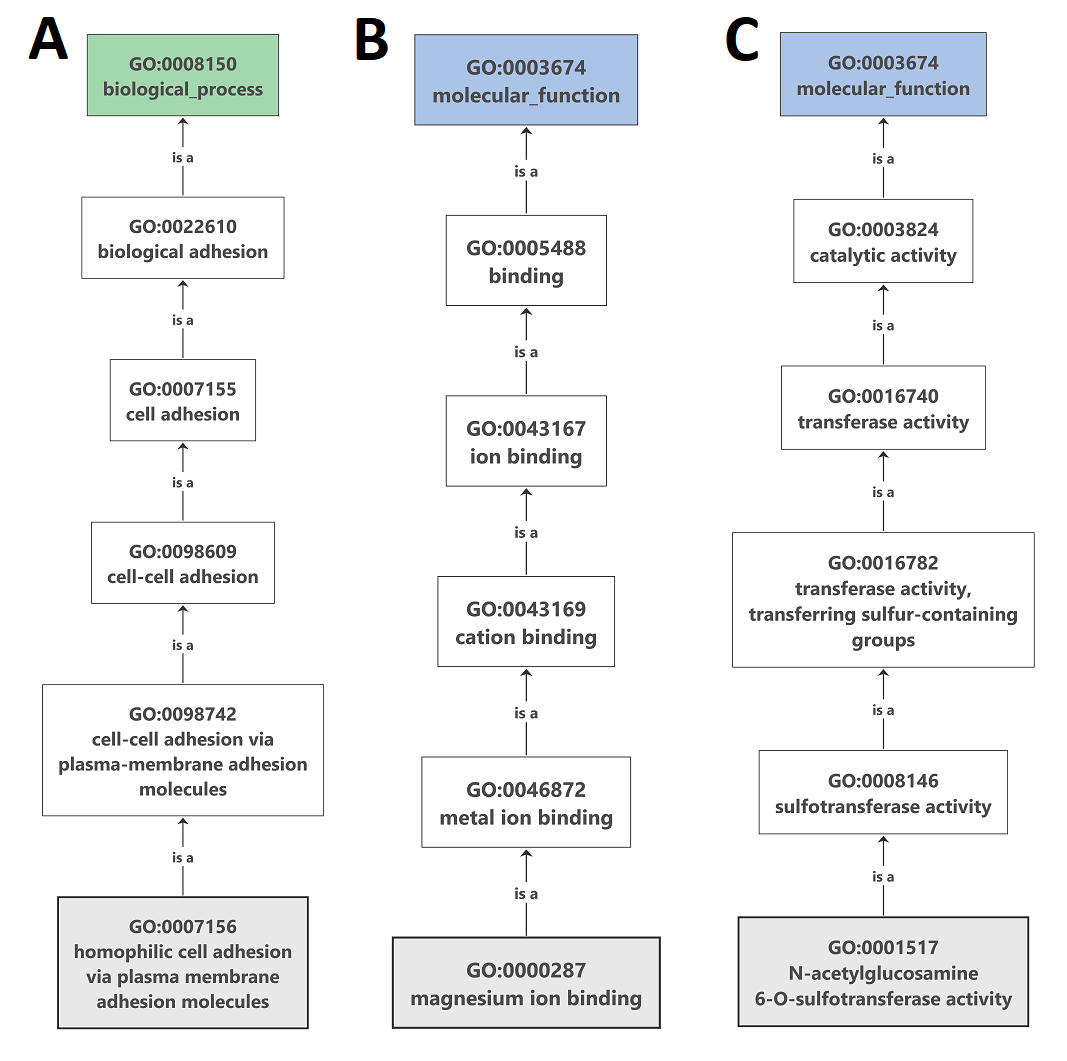

Supplement: Supplementary file 1 [file ijms-22-07810-s001.zip › SupplementaryTables&Figures/Supplementary_Figures/SupplementaryFigure 5.tif]

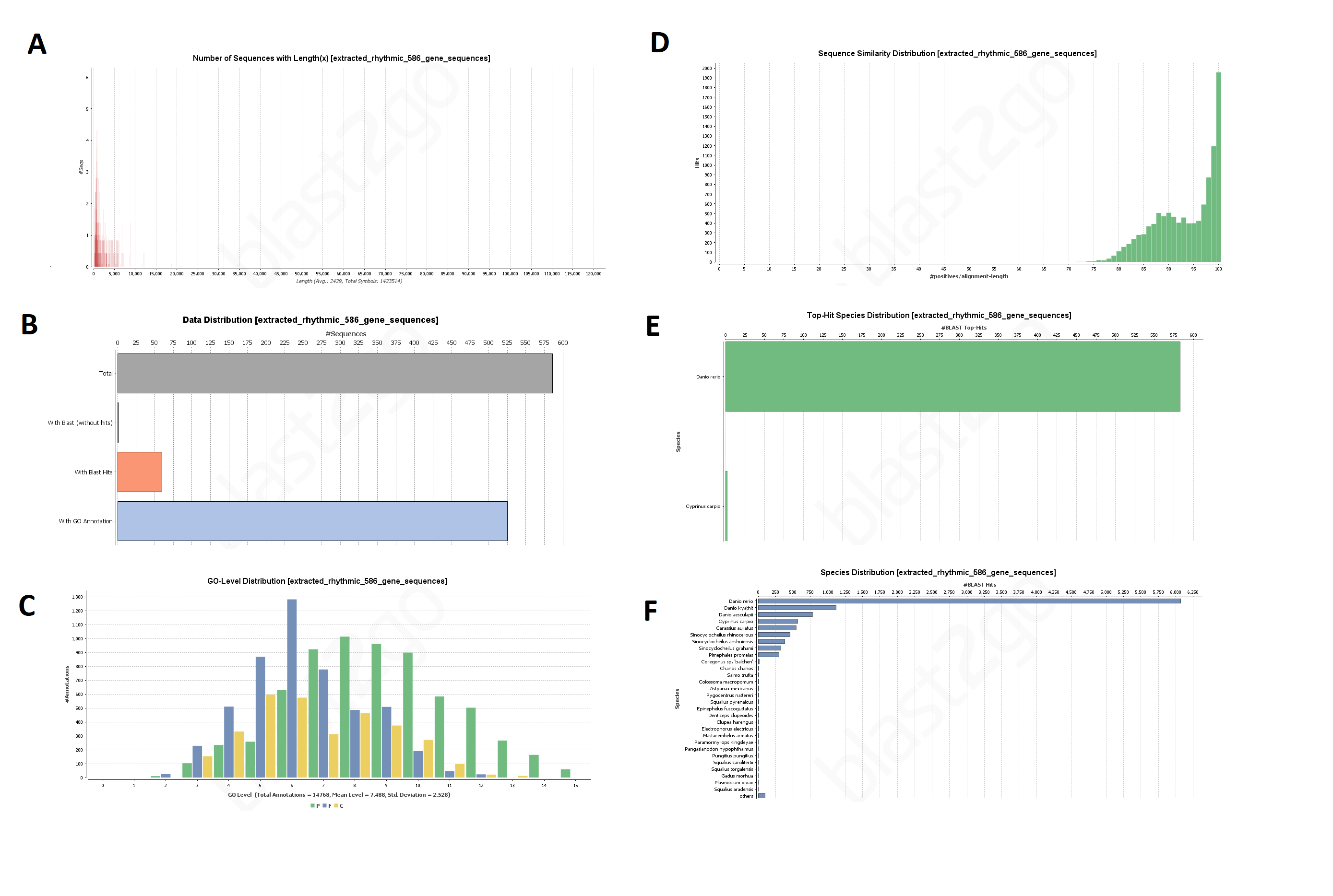

Supplement: Supplementary file 1 [file ijms-22-07810-s001.zip › SupplementaryTables&Figures/Supplementary_Figures/SupplementaryFigure 4.tif]

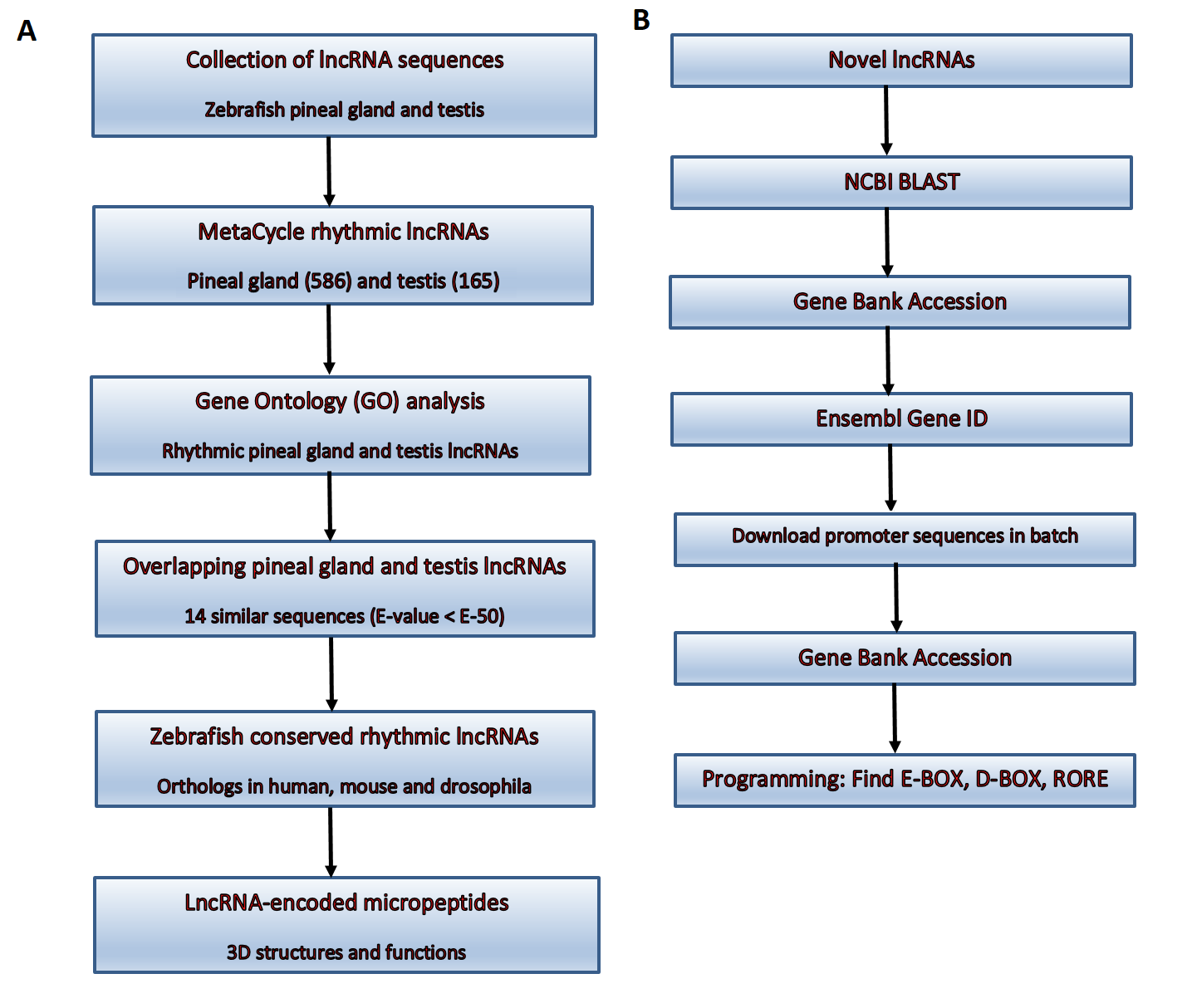

Supplement: Supplementary file 1 [file ijms-22-07810-s001.zip › SupplementaryTables&Figures/Supplementary_Figures/SupplementaryFigure 1.tif]

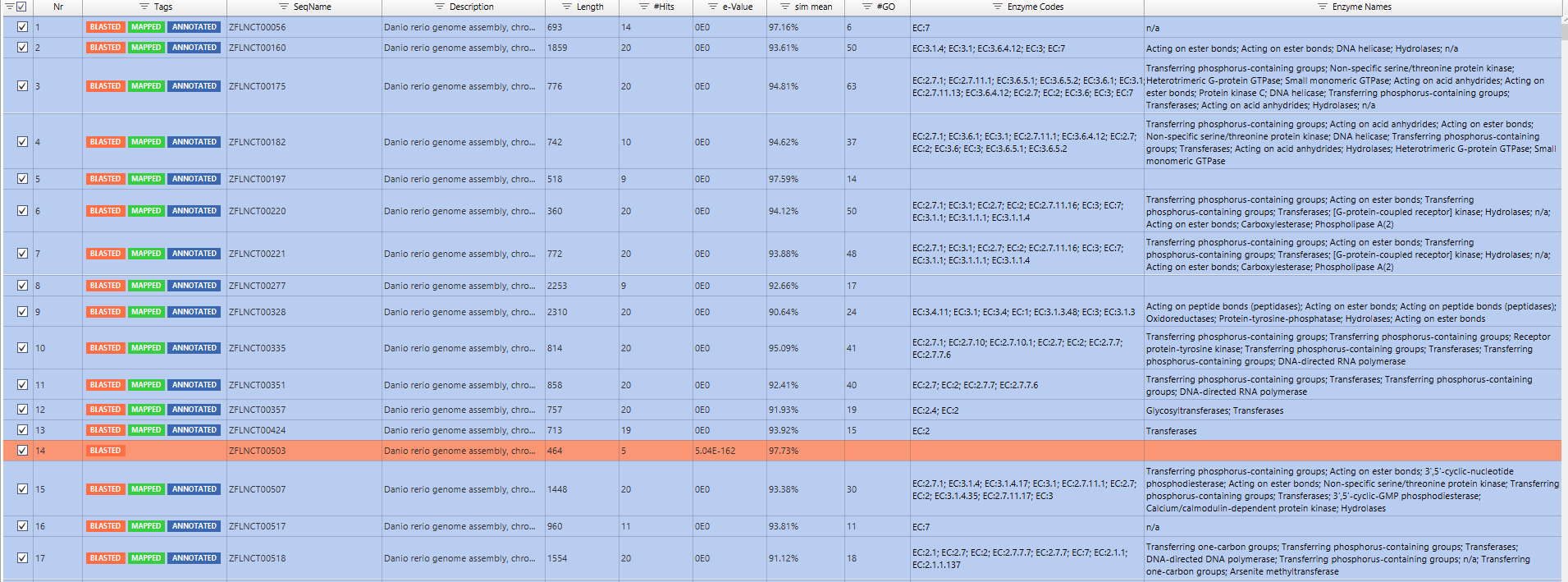

Supplement: Supplementary file 1 [file ijms-22-07810-s001.zip › SupplementaryTables&Figures/Supplementary_Figures/SupplementaryFigure 3.tif]

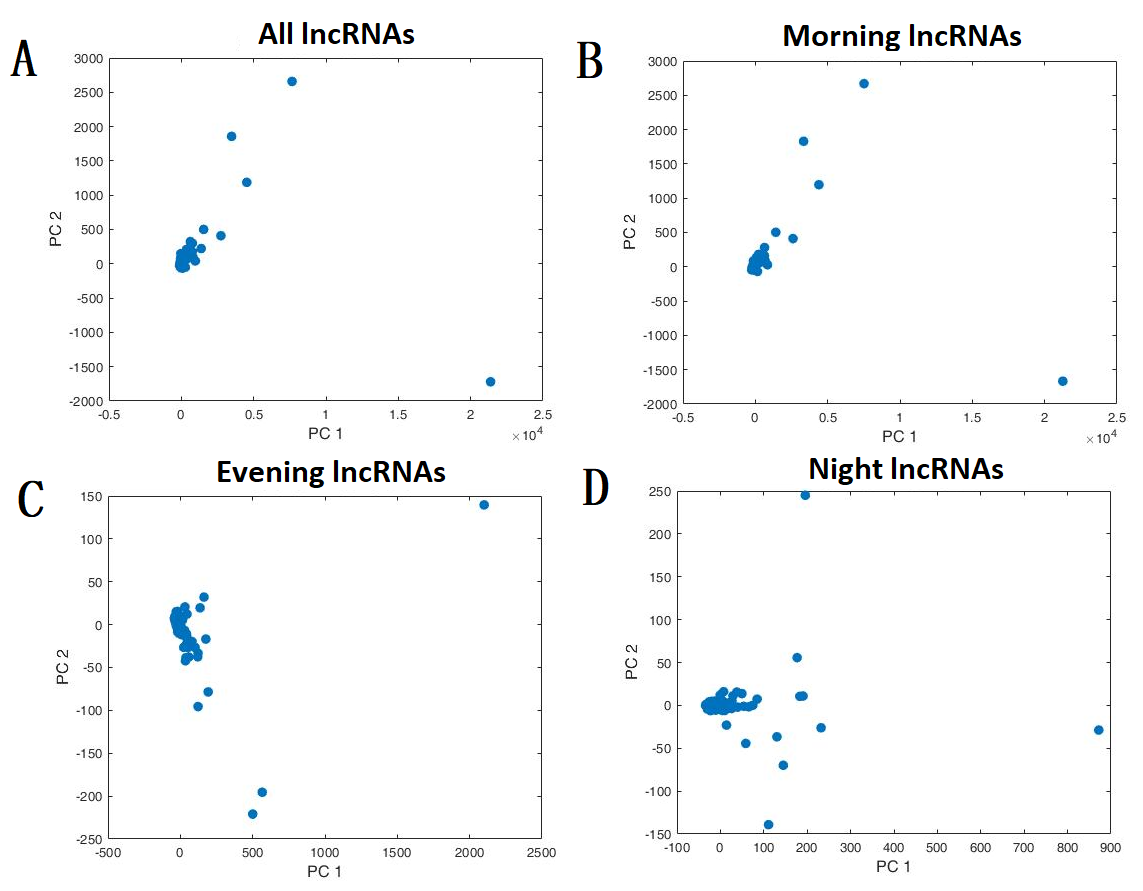

Supplement: Supplementary file 1 [file ijms-22-07810-s001.zip › SupplementaryTables&Figures/Supplementary_Figures/SupplementaryFigure 2.tif]

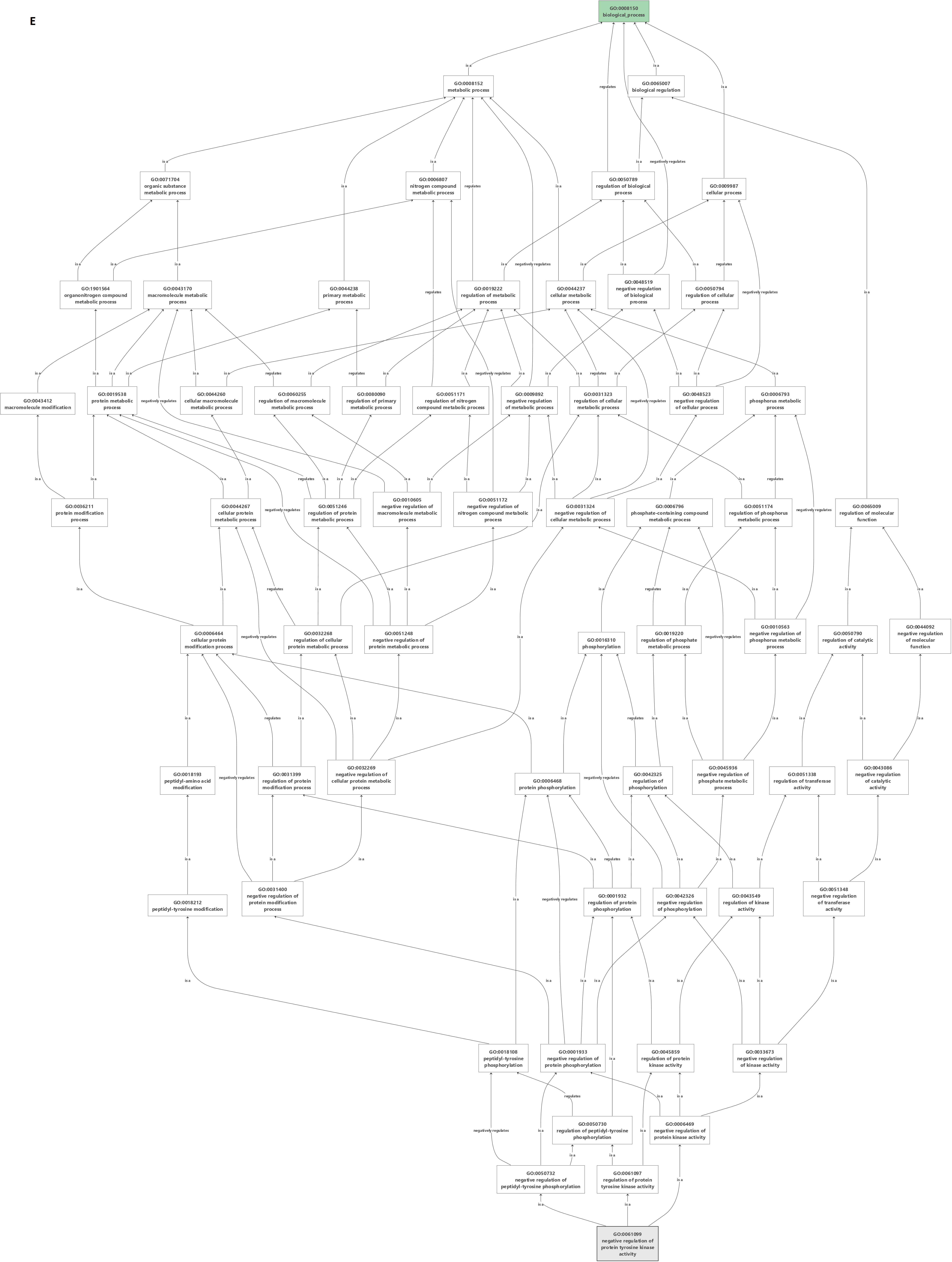

Supplement: Supplementary file 1 [file ijms-22-07810-s001.zip › SupplementaryTables&Figures/Supplementary_Figures/SupplementaryFigure 8.tiff]
